# scHumanNet: a single-cell network analysis platform for the study of cell-type specificity of disease genes

**DOI:** 10.1093/nar/gkac1042

**Published:** 2022-11-09

**Authors:** Junha Cha, Jiwon Yu, Jae-Won Cho, Martin Hemberg, Insuk Lee

**Affiliations:** Department of Biotechnology, College of Life Science and Biotechnology, Yonsei University, Seoul 03722, Republic of Korea; Department of Biotechnology, College of Life Science and Biotechnology, Yonsei University, Seoul 03722, Republic of Korea; Evergrande Center for Immunologic Disease, Harvard Medical School and Brigham and Women's Hospital, Boston, MA, USA; Evergrande Center for Immunologic Disease, Harvard Medical School and Brigham and Women's Hospital, Boston, MA, USA; Department of Biotechnology, College of Life Science and Biotechnology, Yonsei University, Seoul 03722, Republic of Korea; POSTECH Biotech Center, Pohang University of Science and Technology (POSTECH), Pohang 37673, Republic of Korea

## Abstract

A major challenge in single-cell biology is identifying cell-type-specific gene functions, which may substantially improve precision medicine. Differential expression analysis of genes is a popular, yet insufficient approach, and complementary methods that associate function with cell type are required. Here, we describe scHumanNet (https://github.com/netbiolab/scHumanNet), a single-cell network analysis platform for resolving cellular heterogeneity across gene functions in humans. Based on cell-type-specific gene networks (CGNs) constructed under the guidance of the HumanNet reference interactome, scHumanNet displayed higher functional relevance to the cellular context than CGNs built by other methods on single-cell transcriptome data. Cellular deconvolution of gene signatures based on network compactness across cell types revealed breast cancer prognostic markers associated with T cells. scHumanNet could also prioritize genes associated with particular cell types using CGN centrality and identified the differential hubness of CGNs between disease and healthy conditions. We demonstrated the usefulness of scHumanNet by uncovering T-cell-specific functional effects of *GITR*, a prognostic gene for breast cancer, and functional defects in autism spectrum disorder genes specific for inhibitory neurons. These results suggest that scHumanNet will advance our understanding of cell-type specificity across human disease genes.

## INTRODUCTION

Genes do not act in isolation, because the proteins they encode interact with each other and with other molecules. From the perspective of network biology, molecular interactions determine the function of each cell type (1). However, cell-type-specific molecular interactions are difficult to identify and interpret due to context dependency. The advent of single-cell RNA sequencing (scRNA-seq) has enabled the characterization of distinct cell types from complex tissues, as well as the determination of their interactions within mixed-cell populations (2).

A major difficulty in cell-type-specific gene network (CGN) inference from single-cell transcriptome data is the lack of a gold standard for cell-type-specific gene interactions. Accordingly, researchers often use simulated synthetic networks (3). An evaluation using reference protein-protein interactions showed that most methods for network inference, including those developed for bulk RNA-seq data and scRNA-seq, were not capable of reconstructing accurate networks of gene interactions from scRNA-seq data (4). This poor performance is likely due to elevated sparsity (5) and spurious technical variation (6) among scRNA-seq data. To overcome this problem, an accurate network modeling method that uses scRNA-seq data to study cell-type-specific gene functions should be developed.

Two approaches to network construction using single-cell transcriptome data exist: reference-free and reference-guided inference. The former, which is more popular, enables the discovery of gene interactions directly from single-cell transcriptome data, but it suffers from a generally high false-positive rate (4,7). In contrast, the reference-guided approach builds a network by filtering the reference interactome for a given transcriptome of context-associated single cells. Filtered interactions are highly likely to exist in a given cell type.

Here, we describe scHumanNet, a computational platform for the reference-guided construction of CGNs using single-cell transcriptome data. As the reference interactome, we used HumanNet (8), one of the best-performing human gene networks for disease gene predictions. We utilized a modified version of the SCINET algorithm (9). Along with CGN construction, scHumanNet provides several analytical tools to aid the study of cell-type-specific effects of disease genes. Through network centrality analysis, we found that scHumanNet outperformed other single-cell network inference methods in retrieving cell-type-specific genes, suggesting it was suitable for the study of gene cell-type specificity. We demonstrated that genes relevant to the same cell type showed higher within-group connectivity (i.e. compactness) within the network. Utilizing network compactness across CGNs, we deconvolved breast cancer prognostic signatures into cell types and identified those associated with immune cells rather than cancer cells. We also found that the prognostic value of a known signature gene, *GITR*, was linked to T cells owing to its T-cell-specific centrality. Furthermore, we developed a statistical framework for differential centrality analysis that revealed cell-type-specific functional defects in disease genes. Applying this analytical framework to brain scRNA-seq data from autism studies, we found elevated dysregulation of the interaction networks in inhibitory and excitatory neurons of disease condition.

## MATERIALS AND METHODS

### Single-cell transcriptome data for network construction

To construct CGNs, we used scRNA-seq data generated from biopsy samples of breast, lung, colorectal, and ovarian cancers with cell type annotations obtained from Qian *et al.* (10). For pan-cancer comparative network analysis, we focused on five major cell types in the tumor microenvironment: T cells, B cells, myeloid cells, cancer-associated fibroblasts (CAFs), and endothelial cells (ECs). For the study of autism spectrum disorder (ASD), we constructed CGNs for cell types found in the brain using scRNA-seq data obtained from Velmeshev *et al.* (11). The pre-annotated cell types were merged with more granular representations to include ECs, oligodendrocytes, astrocytes (AST-FB and AST-PP), microglia, inhibitory cells (IN-PV, IN-SST, IN-SV2C and IN-VIP), excitatory cells (L2/3, L4, L5/6 and L5/6-CC), and others (Neu-mat, Neu-NRGN-I and Neu-NRGN-II).

### Reference-free CGN construction with single-cell transcriptome data

For network construction, we only considered protein-coding genes defined by the consensus coding sequence (CCDS) database. We built four variants of the co-expression networks, each of them based on each scRNA-seq dataset. In the first co-expression network, we calculated Pearson correlation coefficients (*PCC*) between gene pairs using a count matrix of single-cell transcriptome data, which was log-normalized by the *NormalizeData()* function of the Seurat package. Only links with *PCC* > 0.8 were retained for the rawPCC network. The second type of co-expression network was based on the de-noised count matrix from MetaCell (12,13). To calculate the *PCC* between gene pairs, we used metacells generated with unified threshold and parameters. We discarded cells with fewer than 500 UMIs and used the parameters *K* = 30 and alpha = 2 for *mcell_mc_from_coclust_balanced()*. The third type of co-expression network was based on a count matrix with imputation of dropouts by SAVER (14,15) and exclusion of genes with >99% zero values. The last type of co-expression network was based on data transformation using bigSCale2 (16). The *recursive* method was used for the clustering parameter of *compute.network()*, and the PCC was calculated using the transformed *Z*-score matrix.

The accuracy of co-expression networks based on metacells, SAVER, and bigSCale2 was evaluated using a Bayesian statistical framework and log likelihood score (*LLS*) (17). In this scheme, gold standard gene pairs were used to evaluate the likelihood of data-driven gene pairs such as co-expression links. In brief, for the prioritized gene pairs inferred from the given data (*D*), we calculated *LLS* for every 1,000 links sorted by the data intrinsic score using the following equation:

$$\begin{equation*}LLS\ = \ \left( {\frac{{P\left( {L|D} \right)/P(\neg L|D)}}{{P\left( L \right)/P\left( {\neg L} \right)}}} \right)\end{equation*}$$

where *P*(*L*|*D*) and *P*(¬*L*|*D*) account for the probability of positive and negative gold standard gene pairs in a given dataset, respectively, and *P*(*L*) and *P*(¬*L*) represent the probability of gold standard positive and negative gene pairs, respectively. We used a set of 260 962 gold standard positive gene pairs obtained from HumanNet (8). A set of gold standard negative gene pairs was inferred as being composed of all links not included among gold standard positives.

For the construction of CGNs using GRNboost2 (18), 2416 transcription factors (TFs) gathered from previous publications (19,20) were used as input, and the top 0.1% of links were retained for the final networks.

### Reference-guided CGN construction with single-cell transcriptome data

scHumanNet was developed by modifying the SCINET framework (9) which utilizes imputation, transformation, and normalization of scRNA-seq data. Single-cell gene expression data were pre-processed using the ACTIONet package (21). By identifying the archetypes within the scRNA-seq dataset, ACTIONet learns the dominant transcriptional patterns representative of cell types and states. This approach produces a transformed gene activity score matrix, which is the basis for inferring gene-pair interactions. For each gene pair from the gene score activity matrix, a minimum activity score threshold is applied to assess the strength of the interactions in a group of cells. If each gene in the examined interaction passes the threshold determined by the transformed cell type activity score and a link exists in the reference interactome, it is deemed cell-type-specific and retained in the resultant CGN. Although the SCINET package provides edge weights based on the aggregated *P*-value of a likelihood score, we used the *LLS* from our reference interactome, HumanNet, as the edge weight. The *LLS* for HumanNet edges was also calculated by Bayesian statistics as described above and in Supplementary Methods. We measured the network centrality of each gene based on the sum of *LLSs* to all its neighbors. Because the human interactome is biased towards the ribosome complex (22), we excluded ribosomal proteins from the final candidate hub genes. HumanNet provides three-tire interactome models (8). We have tested all tiers of interactome models and found that the most extensive one, HumanNet-XC, gives best results in general. Therefore, we used the HumanNet-XC for scHumanNet.

### Significance test for network hubness

The statistical significance of hub genes was calculated using the *FindAllHub()* function in the scHumanNet package. For each CGN, randomized networks were generated by swapping edges with equal probability using the *igraph* package function *rewire()*, and the centrality scores of all genes were collected. This process was iterated until at least 10 000 centrality scores were gathered, which were then used to generate a null distribution. By default, Benjamini–Hochberg correction was applied for each *P*-value, and hub genes with false discovery rate (FDR) <0.05 were selected for each CGN.

### Predicting cell-type-specific genes for B and T cells

To test the cell-type relevance of genes, we compiled T- and B-cell-associated genes from the Gene Ontology (GO) database. We used reliable annotations by considering only evidence based on traceable author statement (TAS), inferred from direct assay (IDA), inferred from mutant phenotype (IMP), or inferred from genetic interaction (IGI). By selecting GO term descriptions that contained either ‘T cell’ or ‘B cell’, we obtained 289 genes associated with T cells and 89 with B cells. We conducted a similar compilation for other cell types but could not obtain enough associated genes for statistical testing. We identified differentially expressed genes (DEGs) using the function *FindAllMarkers()* from the Seurat v3.2.3 package with default parameters ‘wilcox’ for *test.use*, ‘0.25’ for *logfc.threshold*, and ‘0.1’ for *min.pct*. We selected protein-coding DEGs with positive log-fold changes for B or T cells (*q*-value < 0.05) as cell-type-specific genes. Finally, we measured the weighted degree centrality of genes using the sum of edge scores for other network construction methods: *PCC* (rawPCC, MetaCell, SAVER and bigSCale2), importance score (GRNboost2), and weighted score (SCINET). Only significant DEGs and hub genes were used to compare cell-type relevance.

### Predicting cell-type-specific TFs

TFs specific for B and T cells were obtained from the TF-Marker database (23) and subsequently filtered using the TRRUST database (24), resulting in 42 T-cell-associated TFs and 14 B-cell-associated TFs. The top 100 hub genes identified by scHumanNet were extracted from each cell type and filtered using the 2416 TFs collated from previous publications (19,20). The top 100 DEGs based on log-fold change values were selected and filtered using the same TF gene list. Hypergeometric tests were performed with all genes in HumanNet (18,593) as the gene space.

### Compactness analysis of gene sets to identify relevant cell types

We implemented the *Connectivity()* function in scHumanNet to evaluate network compactness of a group of genes. Briefly, 10,000 random gene sets with the same number of genes as the test gene set were selected to generate a null model. To preserve the network topological properties for the random gene sets, we used rejection sampling, whereby we selected a gene with ±20% degree of connectivity for each real gene when permuting. Significance was measured using the rank of observed within-group connectivity in the null distribution. Genes that exert their function in a specific cell type tend to be connected to each other in a network specific to the cell type. The degree of compactness was measured using the significance of within-group connectivity. We performed compactness analysis for a set of immune checkpoint molecule (ICM) genes (25) and 33 breast cancer prognostic signature gene sets collected from Huang *et al.* (26). For the GGI97 signature, only 76 out of 97 genes were evaluated in this study because the others had been either discontinued or deprecated in the NCBI gene database (Supplementary Table S1). Their relevance to the cell cycle was assessed using manual curation and accepted databases. Genes that were included in ‘Cell Cycle’ of KEGG 2021, ‘G2-M Checkpoint’ of MSigDB 2020 and ‘Cell Cycle Homo sapiens’ of Reactome DB 2016 were considered cell cycle-related. Other genes were curated manually, and those that included ‘DNA replication’ and ‘mitotic spindle’ were also included. Of the 76 signature genes, 24 were detected in the breast cancer T-cell network and their functional connectivity was assessed through *Connectivity()* with default parameters.

### Survival analysis on The Cancer Genome Atlas (TCGA) breast cancer samples

Only direct neighbors of the *GITR* gene in the T-cell network for breast cancer were considered connected to GGI97 signatures. TCGA data were downloaded through the GDC portal using the *TCGAbiolinks* R package. HTseq counts were preprocessed using *TCGAanlayze_Preprocessing()*, with ‘0.6’ as the *cor.cut* parameter. The data were subsequently normalized using *TCGAanalyze_Normalization()*. The preprocessed count data were normalized with sample-specific size factors calculated using DESeq2. To identify genes indicative of good patient outcomes, we considered 23,192 genes from TCGA-derived expression matrix, of which 1,078 BRCA samples were separated based on the top 30th and bottom 30th percentile of test gene expression. *P*-values from the Kaplan–Meier log test were corrected using the Benjamini–Hochberg method, yielding 236 genes with FDR <0.05, which were regarded as predictive of good prognosis. For survival analysis, samples were separated into high and low groups based on median *GITR* expression. The correlation between *GITR* expression for each bulk sample and the composition of T cells was calculated using the geometric mean of *CD3D*, *CD3E* and *CD3G*. The survival group was divided into high and low groups based on the median of either single gene expression or geometric mean expression of the gene set. Network visualization of the breast cancer T-cell scHumanNet was performed using the *igraph* R package.

### Differential centrality analysis for ASD genes

For each CGN, the degree of centrality was assessed based on the sum of edge weights (*LLS*). Because network size affects the degree of centrality score, we used percentile ranks, whereby the most central gene had a value of 1 and the least central one had a value of 0. We assigned a value of 0 to genes that were not included in at least one of the networks. For each gene, we calculated the differential percentile rank of centrality (diffPR) by subtracting the percentile rank in the control network from the percentile rank in the disease network.

$$\begin{equation*}{\rm{\ }}P{R_{x,N}} = \left\{\begin{matrix} {percentile\ rank\ by\ degree\ centrality\ in\ {N_x}\left( {x \in N} \right)}\\ {0\ \left( {x\ \notin N} \right)} \end{matrix}\right. \end{equation*}$$

$$\begin{equation*}{\rm{\ }}diffP{R_x} = \ P{R_{x,ASD}} - \ P{R_{x,Control}}\end{equation*}$$

where *x* represents a gene and *N* represents a disease or control network for a given cell type.

The percentile rank was calculated using the *dplyr* package *percent_rank()*. The *diffPR* for each gene ranged from -1 to 1, with positive values indicating higher connectivity in the disease network.

For a significance test of differential centrality, we used the *FindDiffHub()* and *TopDiffHub()* functions in scHumanNet. Briefly, *FindDiffHub()* finds a distribution of null *diffPR* values for every gene by random permutation of the control network to measure the significance of the observed differential centrality. Random sampling of *diffPR* values continues until one million random values accumulate. Benjamini–Hochberg correction was applied to calculate the FDR. For *TopDiffHub()*, the *diffPR* of the genes was assessed and filtered for non-zero values. By default, genes within the top 5% of *diffPR* values were selected as differential hub genes. To define lost and gained hub genes in the disease network, 0.7 was set as the threshold. Accordingly, control hub genes with a percentile rank > 0.7 were assessed for their *diffPR* distribution. We observed a clear bimodal pattern dividing the genes around a specific *diffPR* value. Genes with *diffPR* of the same threshold or above (absolute value) were considered as hub genes and were characterized by large changes between healthy controls and disease CGNs. Functional enrichment analysis was performed using the *enrichR* package (27) with pathway terms derived from five pathway databases: Elsevier Pathway Collection (as of March 2022), BioPlanet v.1.0, Reactome 2016, GO Biological Process (GOBP) (as of March 2022), and GO Molecular Function (GOMF) (as of March 2022).

## RESULTS

### scHumanNet effectively retrieves genes specific for each intratumoral cell type

**Figure 1.**
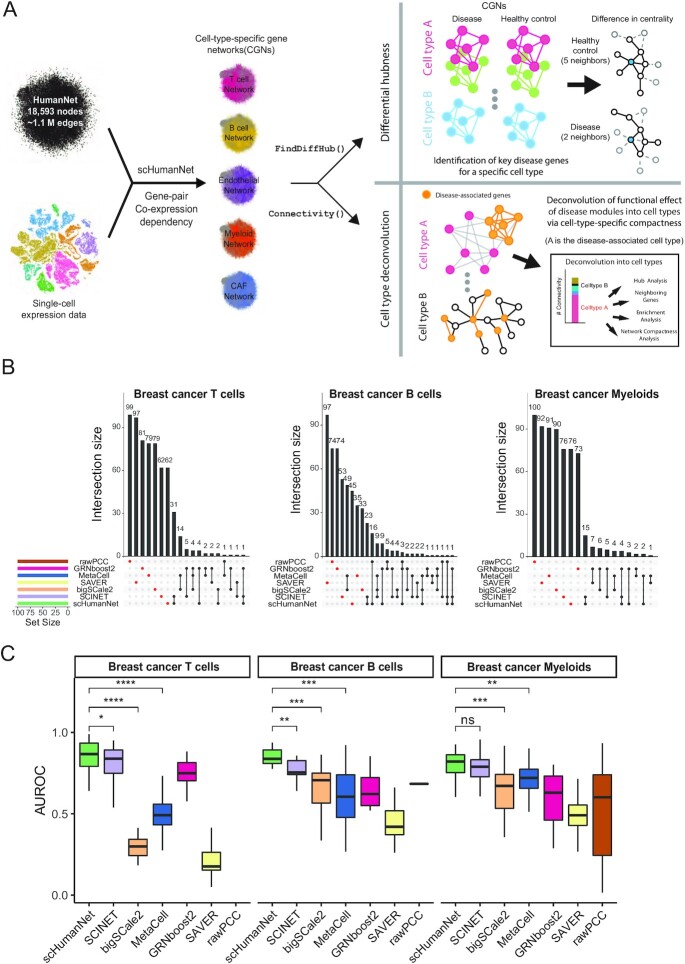
Comparison of cell-type-specific networks generated by scHumanNet and other methods. (**A**) Overview of the scHumanNet platform and downstream analysis scheme used in this study (**B**). Upset plots of the top 100 hub genes in breast cancer networks specific for T cells, B cells, and myeloid cells constructed using seven different network inference methods. (**C**) Area under the receiver operating characteristic curve (AUROC) used to assess retrieval of cell-type-specific genes derived from the Azimuth cell type database by centrality in T, B and myeloid cell-specific networks of breast cancer (all genes, sorted by degree hubness). (*P < 0.05, **P < 0.01, ***P < 0.001 by two-tailed Mann–Whitney *U* test)

To evaluate whether CGNs obtained by scHumanNet (Figure 1A) were more suitable than those generated by other inference methods for the study of cell-type-specific gene functions, we compared various reference-free and reference-guided approaches. Using published breast cancer scRNA-seq data (10), we constructed networks for T cells, B cells, myeloid cells, ECs, CAFs, and cancer cells using five reference-free methods, including rawPCC, MetaCell (12), SAVER (14), GRNboost2 (18), and bigSCale2 (16), as well as one reference-guided method, SCINET (9) based on PCNet (28). Network size across cell types and network inference methods varied widely (Supplementary Table S2). Although GRNBoost2 is a network inference method restricted to TF-target regulatory interactions, we included the method in our comparison because it is the base algorithm for SCENIC (29), a widely applied network modeling method in single-cell biology. The network construction methods compared vary in terms of what types of interactions they consider, and we found that for scHumanNet a wide definition of interactions, which includes evidence based on functional relations, allowed for the best results.

The functional importance of network nodes is measured by their centrality. Cell-type-specific genes presumably play important roles in the corresponding cell types. Therefore, we expected that genes with high centrality values in each CGN were enriched for cell-type-specific genes. Using weighted degree centrality based on the edge scores of each network, we compared the top 100 genes from each network. In this way, we could disregard differences in network size. Interestingly, not much overlap was observed between any of the six network construction methods when assessing the top 100 hub genes for each cell type (Figure 1B, Supplementary Figure S1), and this pattern remained the same with top 50 and 200 hub genes (Supplementary Figure S2) To determine if the hub genes were prioritized for cell-type-specific functions, we assessed the area under the receiver operating characteristic curve (AUROC) score for each cell-type-specific entry. Using the Azimuth celltype database (30), which contains signature marker genes extracted from large scRNA-seq datasets, we observed a higher retrieval rate of cell-type signature genes by centrality in reference-guided CGNs than in reference-free CGNs (Figure 1C). Similarly, the association between the top 100 hub genes and each of the Azimuth cell-type signature genes tended to be stronger in CGNs generated via reference-guided methods than in those that relied on reference-free methods (Supplementary Figure S3). Among reference-guided CGNs, scHumanNet prioritized cell-type-specific genes better than SCINET, especially among B and T cells. These results suggest that scHumanNet is superior to other CGN construction methods in retrieving cell-type-specific functions of human genes. We also found that scHumaNet prediction for cell-type-specific genes is robust against contamination of other immune cell subsets from the same dataset (Supplementary Methods and Supplementary Figure S4).

### scHumanNet reveals commonality and differences among CGNs across cancer types

**Figure 2.**
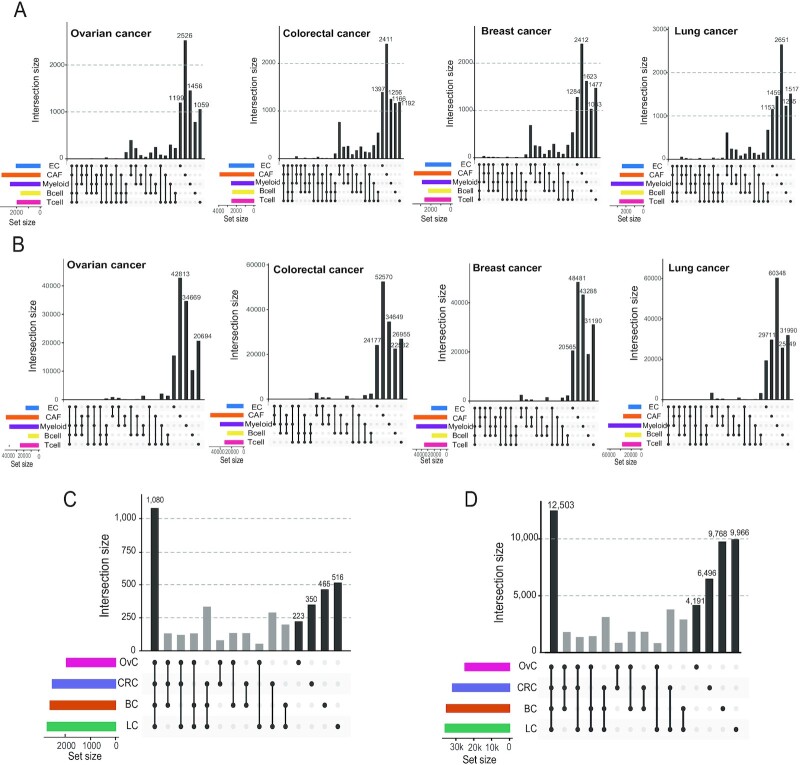
Commonality and differences between CGNs generated by scHumanNet across cancer types. (A, B). Upset plots for five CGNs (T cells, B cells, myeloid cells, CAFs, and ECs) show overlap with respect to nodes (**A**) and edges (**B**) across cancer types. (C, D). Node (**C**) and edge (**D**) overlap of T-cell networks among four cancer types (OvC, ovarian cancer; CRC, colorectal cancer; BC, breast cancer; LC, lung cancer).

The function of tumor-infiltrating cells in cancer is often investigated using cell-type-specific gene expression. Here, we show that network biology can complement expression-based functional studies. To this end, we used scHumanNet to construct CGNs for T cells, B cells, myeloid cells, ECs, CAFs, and cancer cells from breast, colorectal, lung, and ovarian cancers. Next, we examined whether these CGNs could provide functional insights linked to cell type or disease status. Statistics for CGNs relative to each cancer type are summarized in Supplementary Table S3. Network comparisons across different types of non-cancerous cells revealed that only a minor portion of nodes and edges was shared across cell types in all cancers (Figure 2A, B); whereas a large portion was shared across cancer types (Figure 2C, D; Supplementary Figures 5, 6). These results indicate that CGNs generated by scHumanNet are shaped primarily by the cellular context rather than the disease or tissue context. Notably, the ratio of unique edges to shared edges across cancer types was larger than that of unique nodes to shared nodes in all cell types, indicating that networks for the same cell type are rewired in different tissue and disease contexts.

### scHumanNet centrality and compactness predict cell-type specificity of gene functions

**Figure 3.**
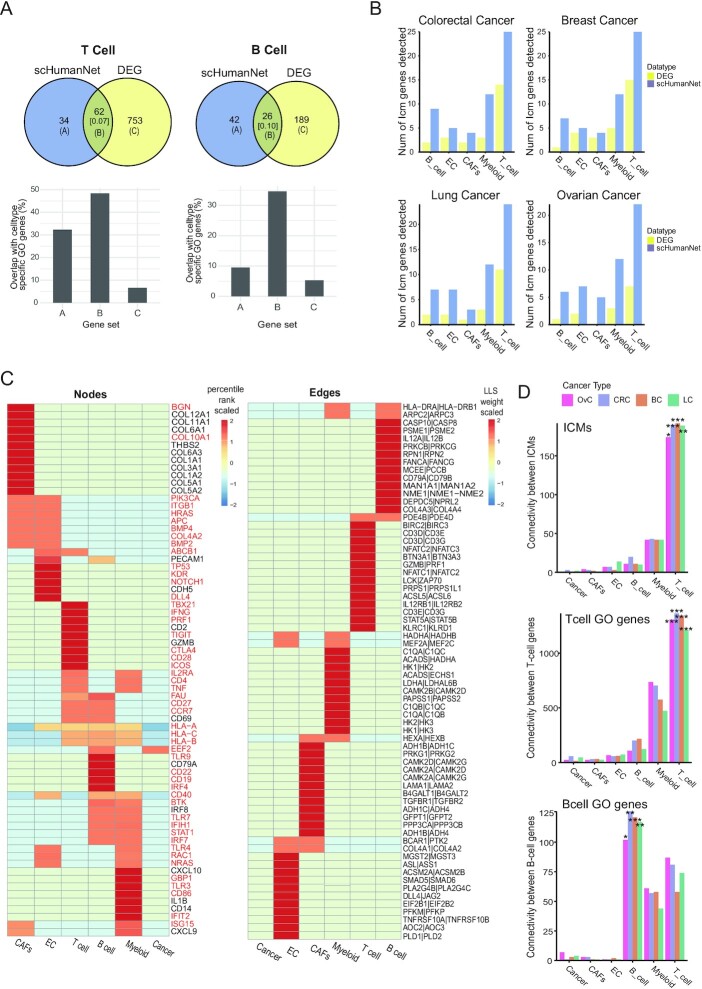
Prediction of cell-type-associated genes via differential expression analysis and network centrality by scHumanNet. (**A**) Overlap of significant DEGs and significant hub genes for B- and T-cell networks in breast cancer (*q*-value < 0.05). The numbers in square brackets correspond to Jaccard indices. Overlap of genes specific for B- and T-cell functions was assessed for prediction by hub genes and DEGs (set A and set C) and their intersection (set B). (**B**) Number of ICMs retrieved via *FindAllMarkers()* (DEG) and *FindDiffHubs()* (scHumanNet) in different cancer types. (**C**) Heat map showing the percentile rank of top 15 hub genes (nodes) and interactions (edges) of each breast cancer network. Values were scaled per row. Results for other cancer types are reported in Supplementary Figure S6. Genes highlighted in red were not among the top 50 DEGs retrieved by the *FindAllMarkers()* function in the Seurat package. (**D**) Within-group connectivity between ICMs, T-cell GO genes, and B-cell GO genes for all annotated cell types in scHumanNet and for each cancer type (OvC, ovarian cancer; CRC, colorectal cancer; BC, breast cancer; LC, lung cancer) (*P < 0.05, **P < 0.01, ***P < 0.001 by non-parametric test)

Rewiring gene interactions in different cell types might change the network centrality of genes with differential functional importance across cellular contexts. Given that hub genes with a high degree of centrality interact with many other genes in a given cellular context, we hypothesized that they were more likely to play important roles in maintaining functions specific to the given cell type. Therefore, we investigated whether scHumanNet hub genes for each type of tumor-infiltrating cell could reflect cell context-dependent functional importance across cancer types. To evaluate cell-type specificity, we utilized the GO database to collate genes reliably associated with either B or T cells (Materials and Methods). Next, we assessed the power of scHumanNet to predict genes specific for each cell type based on overlap with genes known to function in B or T cells. Notably, the network-based and expression-based candidate genes specific for each cell type showed low concordance (0.05–0.13 Jaccard similarity index), indicating complementarity of the two predictions (Figure 3A, Supplementary Figure S7). Moreover, the intersection between the two predictions showed strong overlap with known cell-type-specific genes. Interestingly, for the most part, network-based predictions showed a similar or higher overlap with known cell-type-specific genes than expression-based predictions, further confirming that scHumanNet hub genes could effectively identify cell-type-specific genes.

We anticipated that ICMs would be enriched among genes specific for tumor-infiltrating cells. Hence, we compiled 43 previously identified ICMs (Supplementary Table S4) (25) and compared their overlap with scHumanNet hubs and DEGs across cell types. For all cell types, we observed higher retrieval of ICMs by scHumanNet hub genes than by DEGs (Figure 3B). Notably, in all cancer types and cell types, the ICMs retrieved by DEGs were subsets of those retrieved by scHumanNet (Supplementary Data S1).

We prioritized genes using weighted degree of centrality in the CGNs constructed by scHumanNet and found that it was highly predictive of cell-type-specific hallmark genes (Supplementary Data S2). Based on this observation, we chose to more closely investigate TFs, which are key determinants for the differentiation and maintenance of particular cell identities. Cell-type-specific differential expression analysis is often insufficient to detect TFs for a given cell type because of a generally low basal level of expression. Instead, a network-based approach has been widely used to infer TF-target interactions (29,31). We hypothesized that network centrality in CGNs could effectively prioritize TFs specific for a certain cell type. To evaluate the prediction of TFs specific for cell types by DEGs and scHumanNet centrality, we retrieved cell-type-specific TFs from TF-Marker (23), a manually curated cell-type-specific TF database. Because of the limited number of entries, we could analyze only TFs specific for B and T cells. By comparing the enrichment of known cell-type-specific TFs among the top 100 prioritized genes by scHumanNet centrality with those identified by differential expression, we found that the network-based approach was consistently better at prioritizing TFs in both B and T cells across cancer types (Materials and Methods, Supplementary Data S3).

We also found that scHumanNet centrality could predict cell-type-specific disease-associated genes. For example, the top 15 hub genes in T cells from all types of cancers included those involved in cell-mediated immunity (*GZMB*, *PRF1* and *IFNG*) and immune checkpoint signaling pathways (*TIGIT* and *CTLA4*) (Figure 3C, Supplementary Figure S8, Supplementary Data S2). Notably, four of the five hallmark genes for T-cell immunity (*PFR1*, *IFNG*, *TIGIT* and *CTLA4*) were not found among the top 50 DEGs (Supplementary Data S4). In B cells, *TLR7* and *TLR9* were found to be pan-cancer central genes but were not detected as DEGs. In myeloid cells, the top 50 pan-cancer central genes included seven genes involved in myeloid cell differentiation (*CD4*, *FCER1G*, *IRF8*, *TYROBP*, *TLR2*, *TREM2* and *ITGAM*), but only two of them (*FCER1G* and *TYROBP*) were found among the top 50 DEGs. In CAFs from ovarian cancer, but not from other cancer types, 11 aldehyde dehydrogenase genes (*ALDH1L1*, *ALDH1L2*, *ALDH3A2*, *ALDH1A3*, *ALDH1A1*, *ALDH1A2*, *ALDH2*, *ALDH1B1*, *ALDH4A1*, *ALDH9A1* and *ALDH6A1*) were prioritized in the top 100 hub genes by scHumanNet. Aldehyde dehydrogenase has been associated with poor survival as it promotes tumor growth in ovarian cancer (32). Notably, none of the 11 aldehyde dehydrogenase genes were among the top 100 DEGs in CAFs from ovarian cancer. The *NOTCH1* gene is expressed in ECs, where it promotes metastasis (33). We found *NOTCH1* among the top 20 hub genes in endothelial CGNs for all four cancer types (7th for breast, 13th for colorectal, 11th for lung and 19th for ovarian cancers), but not among the top 200 DEGs in all cancer types. These results suggest that network centrality using scHumanNet can be more effective than differential expression analysis in identifying genes that play important roles in a given cellular context. These results also suggest that *FindAllHubs()* in scHumanNet can identify hub genes with cell-type-specific functions in both healthy and disease contexts.

Rewiring molecular networks across different cell types may result in differential within-group connectivity (or compactness), which can also be used to estimate functional relevance. As a proof-of-concept, we utilized ICM genes and genes specific to B and T cells. The *Connectivity()* function in scHumanNet tests the significance of within-group connectivity against a nonparametric null model using restricted random sampling that does not require the identification of optimal parameters (Methods). As expected, ICM genes and those specific for B and T cells were associated with T-, B- and T-cell types, respectively, in all cancer types (Figure 3D). This suggests that the network-based approach provides a complementary and intuitive method for assigning gene sets to functionally relevant cell types based on compactness.

### Cell type deconvolution of cancer prognostic signatures using scHumanNet

**Figure 4.**
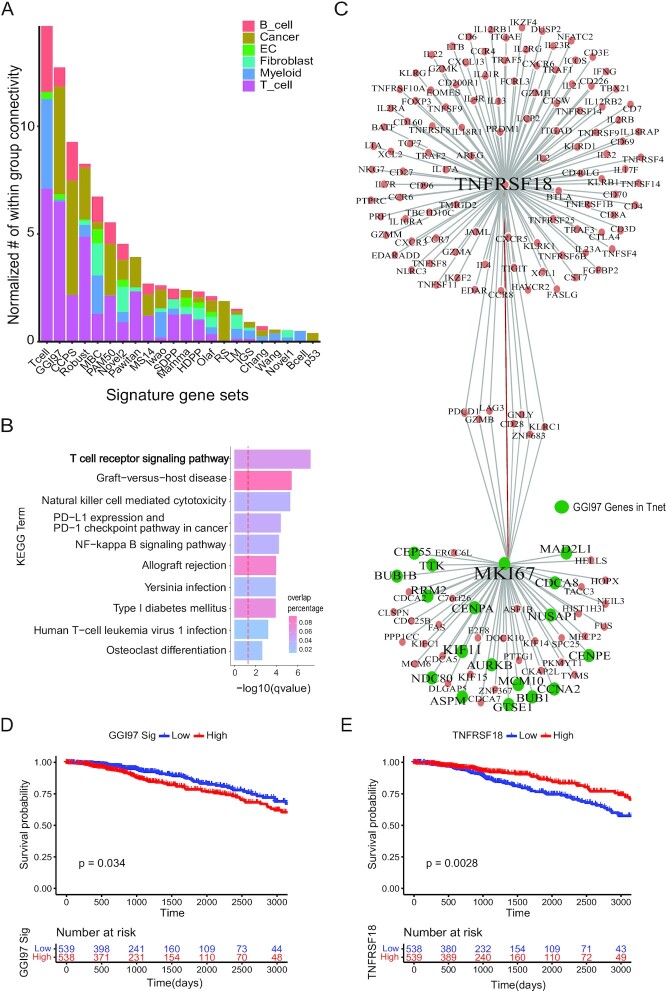
Deconvolution of breast cancer signatures to cell types with scHumanNet. (**A**) Normalized within-group connectivity of each breast cancer signature in six cell-type-specific networks by scHumanNet. Within-group edge counts were normalized to the number of genes for each cell-type-specific network. Cancer signatures with at least 10 genes are presented. (**B**) Gene set enrichment analysis with the KEGG pathway for the top 30 direct neighbor genes of GGI97 signature genes. The red vertical line corresponds to a *q*-value of 0.05 corrected with the Benjamini–Hochberg method. (**C**) Network of genes neighboring *MKI67* and *GITR* (*TNFRSF18*) in the context of breast cancer T cells by scHumanNet. Green nodes denote GGI97 genes. (**D, E**) Kaplan–Meier plot for TCGA-BRCA cohort based on the average expression of 76 signature genes (**D**) or the expression of *GITR* (*TNFRSF18*) (**E**). Clinical samples were divided into high and low groups by median value.

ICMs showed the highest compactness in the T-cell network, which is consistent with their cellular role. We hypothesized that we might deconvolve disease-associated gene signatures obtained from bulk tissues into individual cell types using their network compactness across CGNs by scHumanNet. For example, cancer prognostic signatures are presumably associated with cancer cells because they are typically identified in tumor tissues. However, tumor tissues often contain also non-cancerous cells, such as stromal and immune cells, and some prognostic genes may exert their functions in non-cancerous cells of the tumor microenvironment. To test this hypothesis, we examined 33 prognostic signatures reported in breast cancer (26). We measured the normalized within-group-connectivity of each prognostic signature across the CGNs using scHumanNet (Figure 4A). As expected, we observed strong network compactness for many prognostic signatures from non-cancerous cells, particularly from T cells for the ‘T-cell metagene signature’ (Tcell) (34), ‘97-gene genomic grade index’ (GGI97) (35), ‘127-gene classifier’ (Robust) (36), and ‘64-gene expression signature’ (Pawitan) (37). These results indicate that T-cell function may in part account for the clinical outcomes of breast cancer.

Next, we focused on the GGI97 signature (Supplementary Figure S9A), which has been extensively studied and clinically validated to have an inverse correlation with survival and a positive association with chemotherapy response (38). GGI97 genes were mostly associated with the cell cycle and G2/M checkpoint pathways (47/76 genes) (Supplementary Data S5). Additionally, the 24 GGI97 genes detected in the T-cell network were closely connected to each other (*P* = 0.0001) (Supplementary Figure S9B, C) and significantly enriched in cell cycle-related functions (*P* = 0.0075 by hypergeometric test) (Supplementary Table S1), suggesting a role for T cell cycle control in antitumor activity. We also found that many GGI97 genes were connected to genes with a high degree of centrality and important for T cell antitumor activity (*GZMB*, *PDCD1*, *KLRC1*, *TNF* and *ICOS*) (Supplementary Figure S9D). In particular, genes directly connected to GGI97 signature genes were enriched in the T cell receptor signaling pathway (Figure 4B), indicating that a high GGI97 score primed the immune system for a better response to chemotherapy (39).

T cell proliferation is important in the immunotherapy response. Out of 24 GGI97 signature genes in the T-cell network, 18 were direct neighbors of *Ki67* (Figure 4C), a known marker of cell proliferation. The GGI97 signature is associated with poor survival, which was confirmed by the median expression of GGI97 genes in TCGA-BRCA samples (Figure 4D). To understand the role of GGI97 genes in T cells, we examined the top 10 hub genes directly connected to GGI97 genes in the T-cell network. Notably, *GITR* (*TNSFR18*), a hub gene directly connected to *Ki67*, was prognostic of positive clinical outcomes (Figure 4E, Methods). Importantly, the expression of *GITR* did not correlate with the abundance of T cells (Methods), ensuring that we observed the cellular effect of *GITR* regardless of T-cell composition in each tumor sample (Supplementary Figure S9E). *GITR* has a co-stimulatory role (40) which is essential for CD8^+^ T cells to mount an antitumor immune response. When T cells bind to the ligand *GITRL*, *GITR* promotes the proliferation of effector T cells and dampens the suppressive activity of regulatory T cells (41). The GGI97 signature is predictive of chemotherapy responses. Chemotherapy can promote the cancer-immunity cycle by releasing neoantigens from dead cancer cells. Thus, the beneficial effect of *GITR* can be explained in terms of antitumor immunity. Moreover, we believe that the prognostic effect of the GGI97 signature in chemotherapy is tied to T-cell function via *GITR*. Consistent with our results, *GITRL* combined with anti-PD1 immunotherapy was shown to be effective against breast cancer, resulting in enhanced T-cell activation, proliferation, and memory differentiation (42). Taken together, our findings demonstrate that scHumanNet can deconvolve cancer prognostic signatures into cell types and identify key targets for therapeutic approaches in specific cell types.

### Identification of disease-associated cell types using differential hubness analysis in scHumanNet

**Figure 5.**
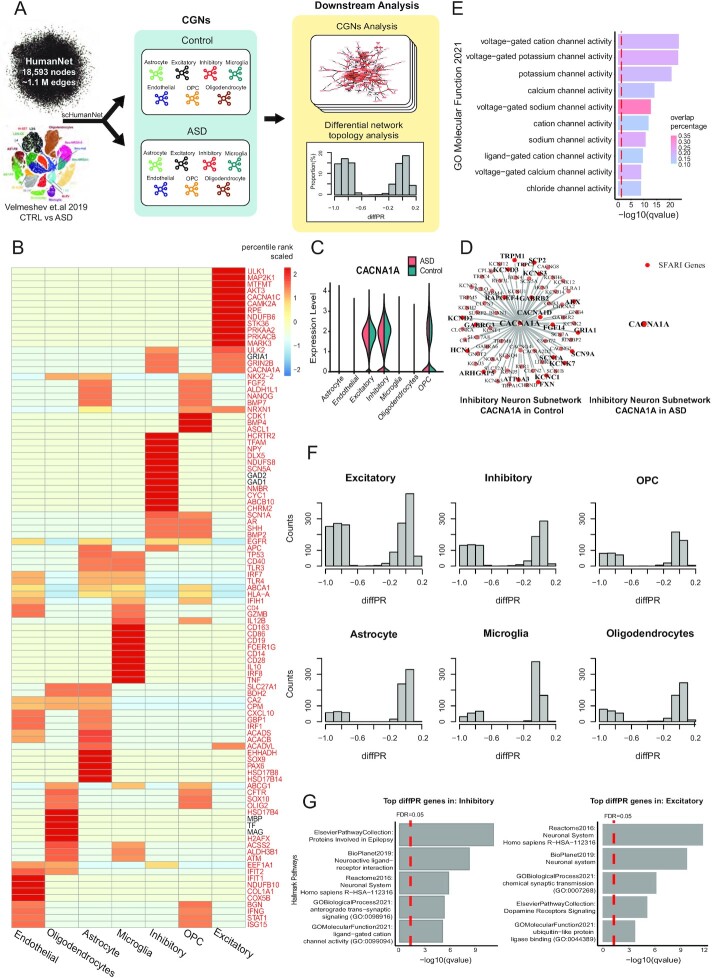
Differential hubness analysis between ASD and healthy control samples across CGNs by scHumanNet. (**A**) Overview of differential hubness analysis by scHumanNet. Seven cell types were grouped and CGNs in normal and ASD conditions were constructed. (**B**) Top 15 hub genes in the combined (control and ASD) networks for seven cell types. Genes highlighted in red were not among the top 50 DEGs identified by the *FindMarkers()* function in the Seurat package. (**C**) Violin plot showing the normalized expression of *CACNA1A* for each cell type in ASD and healthy conditions. The statistical significance of differences between cell types was not evaluated. (**D**) Network visualization of *CACNA1A* and neighboring genes in healthy (left) and ASD (right) inhibitory neurons by scHumanNet. SFARI genes are in red (20 genes out of 72 neighbors in the healthy control, none in ASD). (**E**) Direct neighbors of *CACNA1A* from normal inhibitory neurons by scHumanNet were assessed for enrichment using the GOBP database. The red vertical line corresponds to a *q*-value of 0.05 corrected with the Benjamini–Hochberg method. (**F**) Distribution of diffPR values for genes with hubness (PR) > 0.7 in control cell types. (**G**) Hallmark pathways of genes in ASD derived from five pathway databases (Reactome, BioPlanet, Elsevier Pathway Collection, GO Biological Process, GO Molecular Function) and identified in inhibitory neurons (left) and excitatory neurons (right).

Another application of scHumanNet is the identification of differential hubs, that is, genes whose centrality changes significantly between two biological contexts, such as disease and healthy conditions. The *FindDiffHub()* function in scHumanNet assigns ranks to the genes based on the degree of centrality in each context-specific network, and then identifies those genes whose percentile rank has changed significantly compared to a null model. In addition, the *TopDiffHub()* function allows users to extract the top *n* differentially ranked genes (Materials and Methods). Using differential hubness analysis with scHumanNet, we investigated ASD, a neurodevelopmental disorder with strong heritability (43). ASD is characterized by difficult social interaction and communication, repetitive behavior, and/or sensory susceptibility, and is likely to have many different genetic and environmental causes. A large cohort study by the SFARI consortium identified 1231 genes (44). However, the mechanisms of action of most genes remain poorly understood. We hypothesized that, in the disease condition, perturbation of SFARI genes could result in cell-type-specific loss of wild-type molecular interactions. Thus, a decrease in network centrality could point to disease-associated cell types. Using a published dataset containing 104 559 cells from 15 donors diagnosed with ASD and 16 matching controls (11), we constructed seven CGNs for both healthy and disease conditions (Figure 5A, Supplementary Table S5, Materials and Methods). We found that the scHumanNet hub genes for each cell type were relevant to cell-type-specific functions (Figure 5B). For example, the NMDA receptor subunit *GRIN2B* is a hub gene in both excitatory and inhibitory neurons, and the TF *SOX9* is a hub gene in astrocytes (45). We also observed *CD163*, *FCER1G* and *CD14* as hub genes in microglia (46). Interestingly, unlike the immune cell dataset, whereby a few hub genes were also detected as marker genes via DEGs, most hub genes of the brain scHumanNets were not prioritized via differential expression analysis (Supplementary Data S6) and, indeed, showed minimal overlap with cell-type-specific DEGs (Supplementary Figure S10).

Our analysis also revealed that many genes differed significantly in terms of network centrality between the control and disease conditions, despite modest fold changes (Supplementary Figure S11A). By assessing genes with the highest differences in centrality rank via *FindDiffHub()* with default parameters (Methods), we found that differential hubs from excitatory and inhibitory neurons were significantly enriched with SFARI genes, which contrasted with DEGs being found mostly in ECs and astrocytes (Supplementary Data S7, S8). In particular, the highest overlap between differential hub genes and SFARI genes was observed in excitatory neurons (Supplementary Data S8), although several key ASD genes, including *GRIN2B* and *MECP2* (47,48), were found as differential hubs in inhibitory neurons. Even though *GRIN2B* and *MECP2* are expressed in both excitatory and inhibitory neurons, they were found to be differential hubs only in the latter (Supplementary Figure S11B), implying that they may be functionally more important in inhibitory neurons. This finding has been experimentally validated in a mouse model (49) and suggested by a human study (50), in which inhibitory neurons were enriched for overexpressed SFARI genes. Similarly, for *CACNA1A*, we found that although it was not differentially expressed in inhibitory neurons (Figure 5C), there was a significant difference in terms of network centrality (Figure 5D), and many of the functional interactions were lost in the ASD inhibitory neuron network. The interacting genes were mostly associated with ion channels (Figure 5E), suggesting that the function of neural regulation, especially in inhibitory neurons, might be impaired by *CACNA1A* loss-of-function mutations (51). These results demonstrated that differential hubness analysis using scHumanNet could reveal disease-associated cell types.

Finally, we investigated whether genes with high centrality in healthy conditions but low centrality in disease conditions might provide insights regarding cell-type-specific disease mechanisms (Methods). We found that excitatory neurons, inhibitory neurons, and oligodendrocyte progenitor cells had the highest frequency of loss-of-function genes compared to other cell types (Figure 5F). Notably, genes with high centrality in disease but low centrality in healthy controls were less frequent across all cell types (Supplementary Figure S12A). Gene set enrichment analysis of hubs lost in neurons revealed that their function was primarily associated with neuronal activity (Figure 5G). For inhibitory neurons, the hub genes lost under healthy conditions were enriched in ‘increased anxiety-related response’ (MGI Phenotype), ‘anterograde trans-synaptic signaling’ (GOBP), and ‘ligand-gated cation channel activity’ (GOMF). In excitatory neurons, the genes that lost centrality were enriched in ‘chemical synaptic transmission’ (GOBP), ‘dopamine receptors signaling’ (Elsevier Pathway Collection) and ‘protein secretion’ (MSigDB Hallmark). These results imply that, in disease conditions, these hub genes lost most of their interactions with other genes, resulting in the dysregulation of neuronal function in ASD. In contrast, genes that became more central in ASD networks were not enriched in pathways related to neuronal function (Supplementary Figure S12B).

## DISCUSSION

An important goal of single-cell biology is resolving the cellular heterogeneity of human diseases. Single-cell gene expression analysis may enable the identification of disease-associated cell types based on the differential expression of disease-associated genes in specific cell types. In the present study, we described scHumanNet, a computational platform for network-based analysis of cell-type specificity, which can complement expression-based approaches. The core component of this platform is the reconstruction of CGNs, gene network specific to distinct cell types. Single-cell transcriptome data have been utilized to construct CGNs with either reference-guided or reference-free network inference methods. The evaluation of inferred CGNs is not a trivial task because of the lack of high-quality and experimentally validated gene-gene interactions for particular cell types. In fact, because of the high false positive rate of inferred gene-gene interactions from single-cell transcriptome data, functional hypotheses from these networks are generally based on a group of edges rather than individual ones. Here, we validated the quality of CGNs by the retrieval of cell-type-specific genes among hub genes and network compactness of functional genes in the corresponding cell types. In the present study, we compared various approaches for CGN inference from single-cell transcriptome data and found that reference-guided methods outperformed reference-free methods. These results can be explained by the noisy and sparse nature of single-cell transcriptome data, which generate many false-positive gene-gene interactions (4). Furthermore, among the two reference-guided CGN analysis platforms, scHumanNet was superior to SCINET. Although they utilized the same network inference algorithm, they employed different reference interactomes. Previously, we demonstrated that HumanNet, the reference interactome of scHumanNet, performed significantly better than other human gene networks, including the reference interactome of SCINET, in predicting disease genes (8). This indicates that the quality of the reference interactome is key to the performance of reference-guided CGNs, and future improvement of the former will further ameliorate CGNs.

In this study, we have demonstrated two applications of CGNs in the investigation of cell-type specificity of human disease genes. First, the effects of disease genes can be deconvolved into cell types based on the network compactness of a group of disease genes across CGNs. For example, cell-type deconvolution of breast cancer prognostic signatures showed high compactness not only in cancer cells but also in other tumor-infiltrating cells such as immune cells. The importance of T cells in antitumor activity may account for the large functional bias of prognostic genes towards T cells. Indeed, one of the identified hub genes was *GITR*, a T-cell-specific regulator that plays an important role in the survival of patients with breast cancer. We believe that our network-based approach for associating gene sets with cell types can complement expression-based methods, such as GSVA (52) and scfind (53). In the future, we may expand the scHumanNet platform to systematic cell type deconvolution of disease gene sets for all cell types of each tissue and thus generate CGNs for human cell atlas data. Second, we utilized CGNs to identify disease-associated cell types based on differential hubness between disease and healthy conditions across cell types. Therefore, the scHumanNet platform allows the analysis of differential hub genes. Using the scHumanNet pipeline, we identified inhibitory neurons as a major cell type associated with ASD. These results suggest that a network-based approach can complement an expression-based approach to identify disease-associated cell types using single-cell transcriptome data.

There are some limitations to scHumanNet. Although our results suggest that the reference-guided method yields more biologically relevant CGNs, it comes at the expense of being unable to discover novel interactions specific for the cell type. In addition, cell type deconvolution may be unreliable with a small group of genes (e.g. a set of three genes) because a statistical test for network compactness requires a relatively large number of genes to ensure a sufficient degree of confidence. Further studies are required to address these shortcomings.

In conclusion, we present scHumanNet, a computational platform for single-cell network biology, capable of resolving the cellular heterogeneity of disease-related gene functions. We demonstrate that scHumanNet can deconvolve the functional effect of disease gene sets into cell types and identify disease-associated cell types via topological analysis of CGNs. These results suggest that scHumanNet will boost our understanding of cell-type specificity of human disease genes and thus advance precision medicine.

## DATA AVAILABILITY

The code for scHumanNet and the codes used to generate the figures in this manuscript can be downloaded from https://github.com/netbiolab/scHumanNet.

## Supplementary Material

gkac1042_Supplemental_Files
