# Supplementary material for: scHumanNet: a single-cell network analysis platform for the study of cell-type specificity of disease genes
Source: Nucleic Acids Res. 2022 Nov 9;51(2):e8. doi: 10.1093/nar/gkac1042 (PMC9881140; doi:10.1093/nar/gkac1042)
Supplement: gkac1042_Supplemental_Files [file gkac1042_supplemental_files.zip › Supplementary Methods and Figures.pdf]

Supplementary Methods and Figures

scHumanNet: a single-cell network analysis platform for the study of cell-type specificity of disease genes

Junha Cha, Jiwon Yu, Jae-Won Cho, Martin Hemberg, Insuk Lee

Supplementary Methods

Edge weight score calculation of HumanNet

In scHumanNet we directly utilize the edge weights of the original reference network HumanNet (1) which employs Bayesian statistic framework (2). For the prioritized gene pairs inferred from given data (*D*), we calculated log-likelihood score (*LLS*) for every 1,000 gene pairs using the following equation:

$$LLS = \left(\frac{P(L|D)/P(\neg L|D)}{P(L)/P(\neg L)}\right)$$

, where $P(L|D)$ and $P(\neg L|D)$ account for the probability of positive and negative gold standard gene pairs in given dataset D. $P(L)$ and $P(\neg L)$ represent the probability of gold standard positive and negative links, respectively. The gold standard co-functional pairs were collected from GOBP (as of March 8 2021) (3) and MetaCyc (release 22.5) (4). To avoid bias toward a few pathways with many member genes, we ignored pathways with more than 100 member genes. In addition, we removed super-pathways to avoid bias toward inclusive pathway concepts. As a result, we obtained 260,962 gold standard positive gene pairs (and 38,270,069 negative pairs) among the 8,779 human genes.

Next, we integrated the network with the weighted sum (*WS*) method as validated in previous studies (1,2). For the co-functional gene pairs that are supported by the multiple datasets, the *WS* score is obtained with the following equation:

$$WS = LLS_0 = \sum_{i=1}^{n} \frac{LLS_i}{W \times i}, for\ all\ LLS \geq T$$

, where *LLS* indicates the maximum *LLS* for the gene pairs and LLS_i are sorted *LLS* scores by decreasing order. The weight factor, *W*, and *LLS* threshold, *T*, are optimized for maximizing the area under the plot of *LLS* versus gene coverage.

HumanNet infers co-functional gene pair and gives each pair a probabilistic score through *LLS* scheme from multiple datasets. These datasets include co-citation, protein-protein interaction, pathway database, genetic interaction, gene neighborhood, domain profile, co-expression, phylogenetic profiling, and interlogs. For a more detailed description of how each network was obtained and integrated, refer to the Supplementary Information section of the HumanNet paper (1).

Cell-type-specific gene network (CGN) inference using a reference interactome and single-cell transcriptome data

We utilized a modified version of the SCINET algorithm (5) to infer CGNs with a reference interactome and single-cell transcriptome data. Raw count data from single-cell RNA sequencing (scRNA-seq) analysis were normalized via Archetypal analysis for Cell-Type identification (ACTION) (6) which reduces noise of single-cell expression data by local averaging and smoothing across the cells, while enforcing sparsity constraints on the number of averaged cells to preserve cell state differences. Formally, given an expression matrix S $\in \mathbb{R}^{genes \times cells}$, ACTION reduce the kernel (of dimension cell by cell) to another subspace $S_r \in \mathbb{R}^{D \times cells}$, such that the subspace recapitulates the original kernel matrix. To do this, ACTION computes the SVD decomposition of the orthogonalized expression profile as $S^T_{(ortho)} = \mathbf{U}_r \mathbf{\Sigma}_r \mathbf{V}^T_r$. Then the reduced expression profile $S_r = \mathbf{\Sigma}_r \mathbf{U}^T_r$, so that each row represents metagene and each column a cell. ACTION utilizes a *convex non-negative matrix factorization* (*convNMF*) to optimize for landmark cells by finding the W matrix with the following equation:

$$\min_{\mathcal{K},\mathbf{H}} \left\| \mathbf{S_r} - \mathbf{S_r}(:,\mathcal{K})\mathbf{H}^{(NMF)} \right\|_F^2$$

$$\text{Subject to:} \sum_j h_j^{(NMF)} = 1, 0 \leq h_{ij}^{NMF}$$

, where $h_j^{(NMF)}$ is the jth column of the $H^{(NMF)}$ matrix. With multiple k values (archetypes) combined via running ACTION at multiple resolution, ACTION finds a multi-level archetypal set ($\mathbf{W}^{(M\,L)}$) based on a small number of "close-by" cells. The matrix $\mathbf{H}^{(M\,L)}$ is calculated by regressing over the $\mathbf{W}^{(M\,L)}$ matrix. The W matrix, a profile of metagene × aggregate archetypes, a reverse projection onto the state space of genes is computed using the matrix $\mathbf{V}_r$. For each cell, $\mathbf{H}^{(M\,L)}$ is used to interpolate its corresponding expression profile using the archetypes profile. In brief, the transformed matrix $\hat{\mathbf{S}}$ can be described in a mathematical formula of matrix operation as follows:

$$\hat{\mathbf{s}} = \boldsymbol{V}_r \boldsymbol{W}^{(M\,L)} \boldsymbol{H}^{(M\,L)}$$

This approach is fundamentally different from common gene imputation methods in that signature genes that distinguish cell-types (or archetypes) will have high interpolated expression values, even when their absolute value is small. For a more detailed description of the matrix transformation using ACTION, refer to the Method section of the SCINET paper (5). Of note, scHumanNet does not use the archetypes to infer cell types in the single-cell data (as it has been demonstrated as a core function in the ACTIONet package), but rather gives an input of cell labels as a column in the metadata dataframe (refer to the GitHub tutorial). However, these labels can be derived from either manual curation or automated cell-typing process (e.g. CellTypist (7), ACTION, singleR (8)).

The scHumanNet uses the transformed matrix $\hat{\mathbf{s}}$ to detect cell-type-specific links from its reference interactome, HumanNet. Because different genes have different expression profiles, the transformed matrix is normalized with *rank-based inverse normal transformation*. Briefly, for each gene across cells (row factor), a gene i expression (interpolated) value $\hat{s}_{ij}$ is sorted across all cells and a rank r_{ij} assigned. Then the rank is normalized by the total number of cells, $p_{ij} = \frac{r_{ij}}{n} + 1$. The row-factor matrix $\mathbf{F}^{(r)}$, is then computed by projecting the normalized ranks onto the standard normal distribution: $f_{ij}^{(r)} = -\sqrt{2}\mathrm{erfcinv}(2p_{ij})$, where erfcinv() is the inverse of the complementary error function erfc(), defined as:

$$erfc(x) = \frac{2}{\sqrt{\pi}}\int_x^{\infty} e^{-t^2} dt$$

In a similar fashion, a column factor vector $f_i^{(c)}$, for all gene is defined. Finally, the interpolated expression profile matrix, **T**, is defined in which,

$$t_{ij} = \frac{f_{ij}^{(r)} + f_i^{(c)}}{\sqrt{2}}$$

The co-expression dependencies are assessed by the minimum value of the interacting gene pair. Since each gene expression values have been transformed to follow a standard normal distribution, the right tail of the min operator can be computed using:

$$P(x \leq X) = (1 - \varphi(x))^2$$

Where x corresponds to the minimum transformed expression value of the two genes in a pair, and φ is the cumulative density function (CDF) of the standard normal distribution. While the SCINET algorithm utilize a subsampling scheme to compute the interaction strength (refer to Method section of the SCINET paper), we empirically found that utilizing the reference network probability score benefits the network model by

not assuming gene interaction likelihood based only on the single cell data input. Therefore, while significant HumanNet edges for a specific cell-type is inferred via SCINET algorithm, the edge weight scores of the CGNs are originated from those of HumanNet, which are derived from integration of high-quality multimodal datasets.

Analysis of scHumanNet model robustness against contamination of random cells

To test the robustness of cell-type-specificity of scHumanNet, we artificially introduced cells random selected from those that were not used for network construction as contaminants using a subsampling method as conducted in (9) for the breast cancer dataset. We generated data sets with replacement of 1, 5, 10, 15% of the original cells with randomly selected cells from other immune cell types (cancer cells were excluded). For example, the original T-cell network generated from 14,000 T-cells were compared with a network with 10% contamination that were generated from a population of cells composed of 12,600 of the original T-cells and 1,400 cells from other immune cell types. Area under the receiver operating characteristic (AUROC) curve was calculated using the *ROCit* R package, with the Azimuth database subclassified to relevant celltypes (T cell related, B cell related, and Myeloid related).

Precision-recall analysis is showcased with the breast cancer T cell network, as this cell-type contains the most cell-type-relevant genes from Azimuth Database (187 genes). The top 100 hub genes were used instead of the entire network genes to conservatively measure the precision-recall of the original model compared to the contaminated networks. Network hubness was measured with the sum of weights for directly connected nodes and ranked from 1st to 100th. Within each 100 hub genes of the network based on contaminated data set, precision and recall was calculated using 187 cell-type specific genes. Recall was calculated as the ratio of cell-type specific gene the tested model retrieved compared to the original scHumanNet model. Precision was calculated as the ratio of the model's retrieved hubs within top 100 prioritized genes that are detected in scHumanNet, to all the cell-type specific hub genes the model detected in the top 100 hubs. For AUROC calculation, first, network genes were prioritized by network hubness, which were then followed by all other non-networked genes with random orders. Code to calculate AUROC of the three CGNs and precision/recall of breast cancer T-cell CGN is provided in the scHumanNet github page.

Detecting significant hub genes through network randomization

Assuming that the scHumanNet model reflects cell-type specificity, finding significant hub genes for each network provides valuable information that summarizes the network model. Moreover, network topology comparison between disease and healthy cells may suggest key genes for disease progression via rewired gene-gene interactions. The scHumanNet leverages network randomization to extract statistically significant hub genes in each CGN. To find cell-type-specific hub genes from a CGN, the scHumanNet generates random networks for the CGN by swapping edges with equal probability using the igraph package function *rewire()*. Thus, edge weights become randomly distributed using the *sample()* function. For each random network, we calculate a vector of centralities for all the nodes that exist within the CGN. In *FindAllHubs()*, this process is iterated until 100K random distributions of centrality values are accumulated. Assuming every gene's centrality value follows the same null distribution, we use the same null distribution for every gene within a CGN.

For disease versus healthy network comparison with *FindDiffHub()*, a similar approach is utilized. For each cell-type, both of the healthy and the disease CGNs are randomized. To address computational efficiency we introduce a minimum number of cell threshold (default 500) so that only a sufficiently sized networks are analyzed. For statistical test, we generated null distribution models by collecting centrality scores from randomized networks until one million scores are accumulated. Null distribution is generated once for each CGN that passes the cell number threshold, and *p*-value is calculated for every node within the CGN. We disregard ribosomal protein genes and mitochondrial genes in assessing differential hub genes. For more detailed description, refer to the scHumanNet GitHub repository (https://github.com/netbiolab/scHumanNet).

Supplementary References

1. Kim, C.Y., Baek, S., Cha, J., Yang, S., Kim, E., Marcotte, E.M., Hart, T. and Lee, I. (2022) HumanNet v3: an improved database of human gene networks for disease research. *Nucleic Acids Res*, **50**, D632-D639.

2. Lee, I., Date, S.V., Adai, A.T. and Marcotte, E.M. (2004) A probabilistic functional network of yeast genes. *Science*, **306**, 1555-1558.

3. Gene Ontology, C. (2021) The Gene Ontology resource: enriching a GOld mine. *Nucleic Acids Res*, **49**, D325-D334.

4. Caspi, R., Billington, R., Ferrer, L., Foerster, H., Fulcher, C.A., Keseler, I.M., Kothari, A., Krummenacker, M., Latendresse, M., Mueller, L.A. *et al.* (2016) The MetaCyc database of metabolic pathways and enzymes and the BioCyc collection of pathway/genome databases. *Nucleic Acids Res*, **44**, D471-480.

5. Mohammadi, S., Davila-Velderrain, J. and Kellis, M. (2019) Reconstruction of Cell-type-Specific Interactomes at Single-Cell Resolution. *Cell Syst*, **9**, 559-568.e554.

6. Mohammadi, S., Davila-Velderrain, J. and Kellis, M. (2020) A multiresolution framework to characterize single-cell state landscapes. *Nat Commun*, **11**, 5399.

7. Dominguez Conde, C., Xu, C., Jarvis, L.B., Rainbow, D.B., Wells, S.B., Gomes, T., Howlett, S.K., Suchanek, O., Polanski, K., King, H.W. *et al.* (2022) Cross-tissue immune cell analysis reveals tissue-specific features in humans. *Science*, **376**, eabl5197.

8. Aran, D., Looney, A.P., Liu, L., Wu, E., Fong, V., Hsu, A., Chak, S., Naikawadi, R.P., Wolters, P.J., Abate, A.R. *et al.* (2019) Reference-based analysis of lung single-cell sequencing reveals a transitional profibrotic macrophage. *Nat Immunol*, **20**, 163-172.

9. Sun, D., Guan, X., Moran, A.E., Wu, L.Y., Qian, D.Z., Schedin, P., Dai, M.S., Danilov, A.V., Alumkal, J.J., Adey, A.C. *et al.* (2022) Identifying phenotype-associated subpopulations by integrating bulk and single-cell sequencing data. *Nat Biotechnol*, **40**, 527-538.

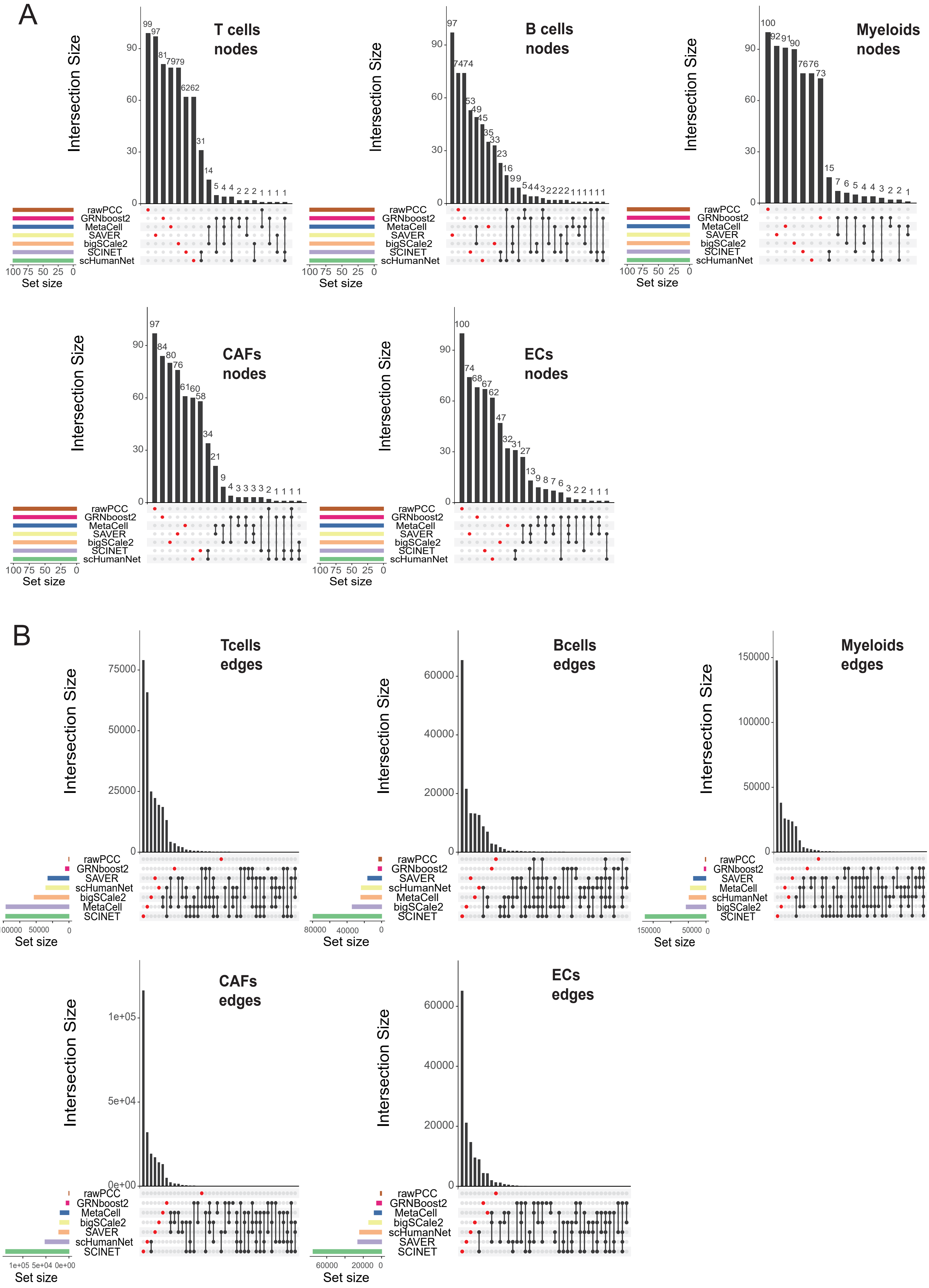
A
T cells nodes
B cells nodes
Myeloids nodes
CAFs nodes
ECs nodes
Intersection Size
Set size
rawPCC
GRNboost2
MetaCell
SAVER
bigSCale2
SCINET
scHumanNet
B
Tcells edges
Bcells edges
Myeloids edges
CAFs edges
ECs edges
GRNBoost2

Supplementary Figure 1. Upset plots for node and edge overlap between cell-type-specific networks (CGNs) by different methods.

A. Upset plots for node overlap. **B.** Upset plots for edge overlap. Five cell types, including B cells, T cells, myeloid cells, cancer-associated fibroblasts (CAFs), and endothelial cells (ECs), were assessed.

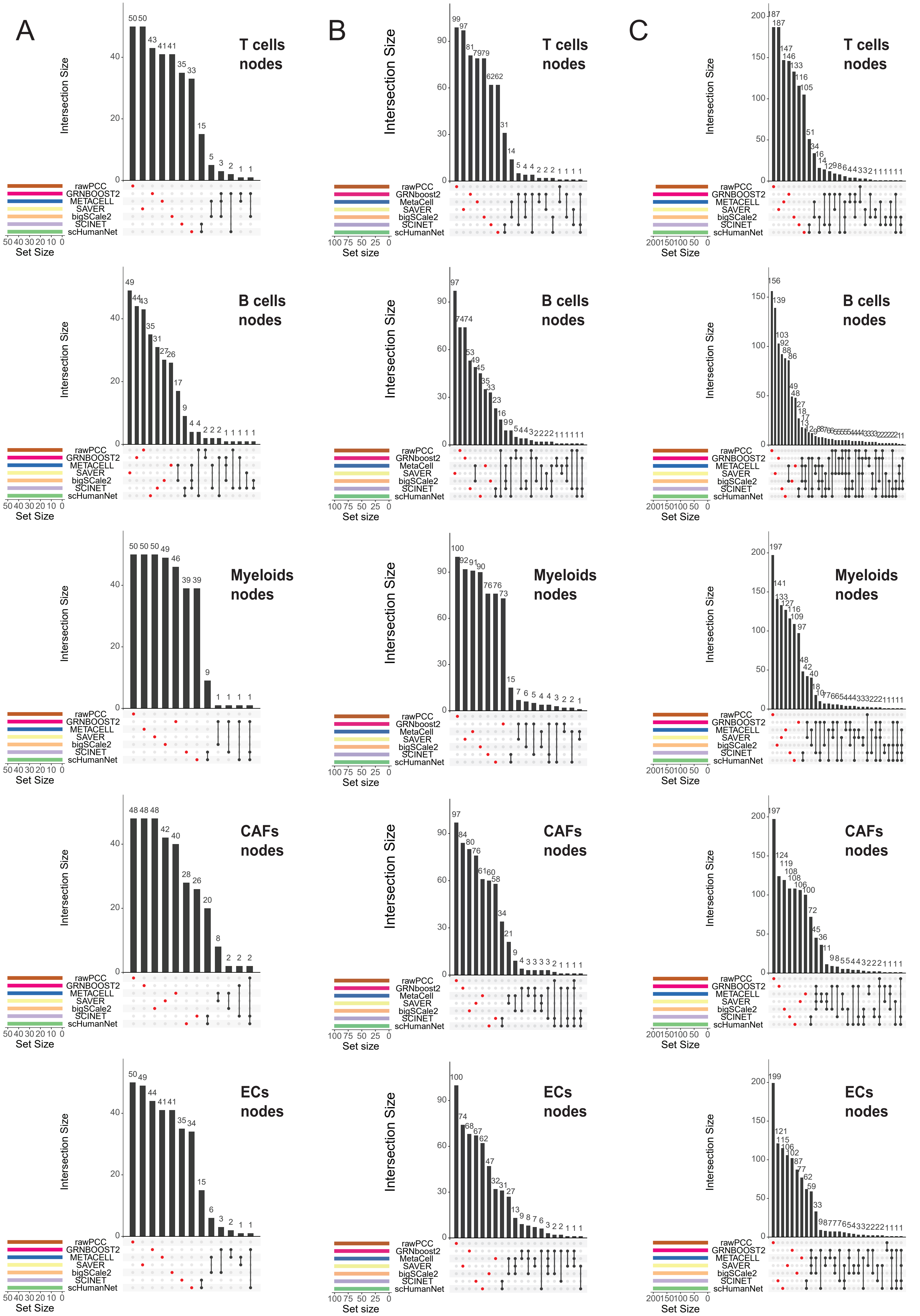
A
B
C
T cells
nodes
B cells
nodes
Myeloids
nodes
CAFs
nodes
ECs
nodes
Intersection Size
Set Size
Set size
rawPCC
GRNBOOST2
GRNboost2
METACELL
MetaCell
SAVER
bigSCale2
SCINET
scHumanNet

Supplementary Figure 2. Overlap of top 50, 100, and 200 central genes constructed from seven different network inference methods. The top 50 **(A)**, 100 **(B)**, and 200 **(C)** hub genes in the networks specific for T cells, B cells, myeloid cells, CAFs, and endothelial cells were assessed for overlap for each different network inference method with upset plot. Unique sets for each method are colored in red dots.

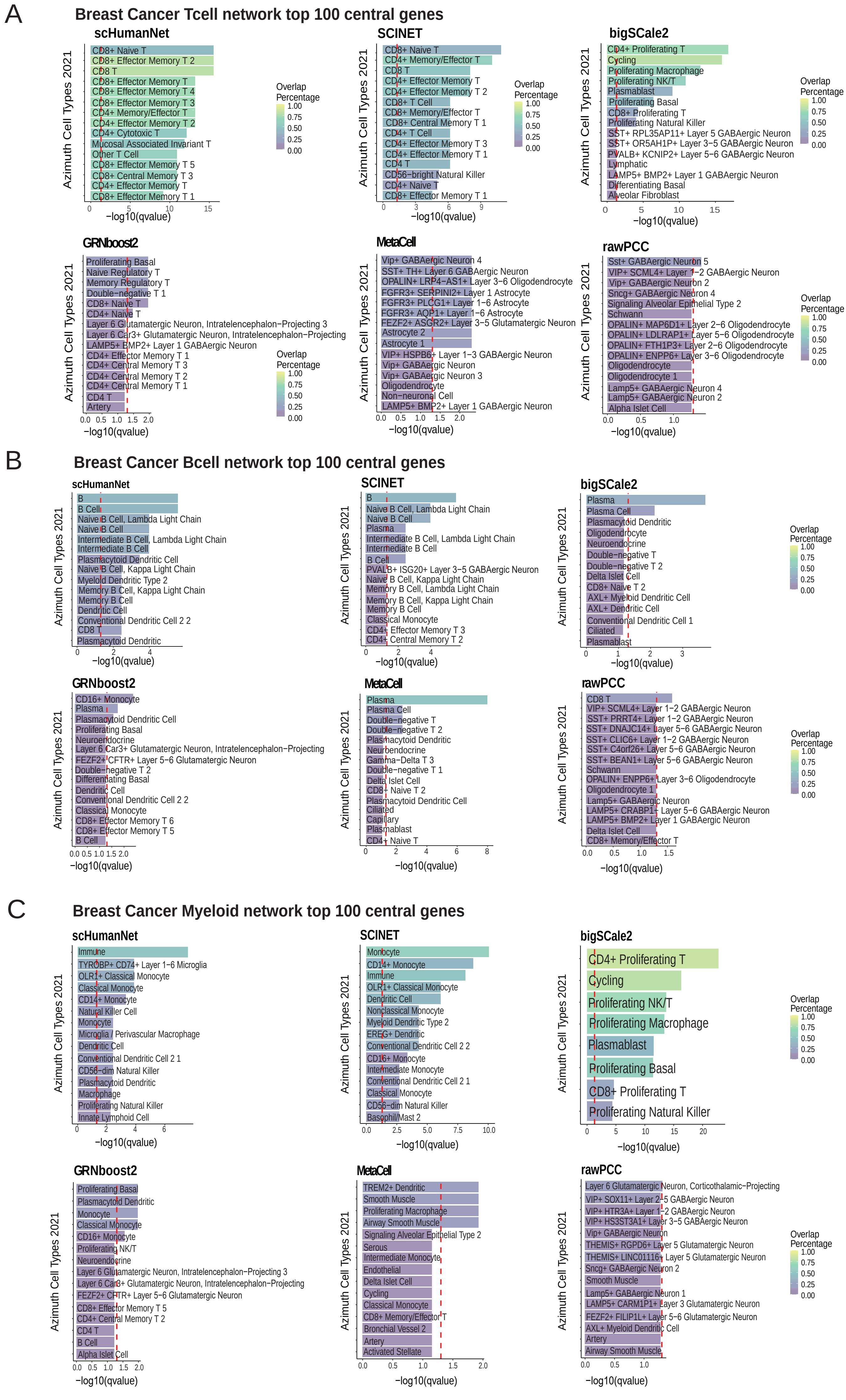

A
Breast Cancer Tcell network top 100 central genes
scHumanNet
CD8+ Naive T
CD8+ Effector Memory T 2
CD8 T
CD8+ Effector Memory T
CD8+ Effector Memory T 4
CD8+ Effector Memory T 3
CD4+ Memory/Effector T
CD4+ Effector Memory T 2
CD4+ Cytotoxic T
Mucosal Associated Invariant T
Other T Cell
CD8+ Effector Memory T 5
CD8+ Central Memory T 3
CD4+ Effector Memory T
CD8+ Effector Memory T 1
SCINET
CD8+ Naive T
CD4+ Memory/Effector T
CD8 T
CD4+ Effector Memory T
CD4+ Effector Memory T 2
CD8+ T Cell
CD8+ Memory/Effector T
CD8+ Central Memory T 1
CD4+ T Cell
CD4+ Effector Memory T 3
CD4+ Effector Memory T 1
CD4 T
CD56−bright Natural Killer
CD4+ Naive T
CD8+ Effector Memory T 1
bigSCale2
CD4+ Proliferating T
Cycling
Proliferating Macrophage
Proliferating NK/T
Plasmablast
Proliferating Basal
CD8+ Proliferating T
Proliferating Natural Killer
SST+ RPL35AP11+ Layer 5 GABAergic Neuron
SST+ OR5AH1P+ Layer 3−5 GABAergic Neuron
PVALB+ KCNIP2+ Layer 5−6 GABAergic Neuron
Lymphatic
LAMP5+ BMP2+ Layer 1 GABAergic Neuron
Differentiating Basal
Alveolar Fibroblast
GRNboost2
Proliferating Basal
Naive Regulatory T
Memory Regulatory T
Double−negative T 1
CD8+ Naive T
CD4+ Naive T
Layer 6 Glutamatergic Neuron, Intratelencephalon−Projecting 3
Layer 6 Car3+ Glutamatergic Neuron, Intratelencephalon−Projecting
LAMP5+ BMP2+ Layer 1 GABAergic Neuron
CD4+ Effector Memory T 1
CD4+ Central Memory T 3
CD4+ Central Memory T 2
CD4+ Central Memory T 1
CD4 T
Artery
MetaCell
Vip+ GABAergic Neuron 4
SST+ TH+ Layer 6 GABAergic Neuron
OPALIN+ LRP4−AS1+ Layer 3−6 Oligodendrocyte
FGFR3+ SERPINI2+ Layer 1 Astrocyte
FGFR3+ PLCG1+ Layer 1−6 Astrocyte
FGFR3+ AQP1+ Layer 1−6 Astrocyte
FEZF2+ ASGR2+ Layer 3−5 Glutamatergic Neuron
Astrocyte 2
Astrocyte 1
VIP+ HSPB6+ Layer 1−3 GABAergic Neuron
Vip+ GABAergic Neuron
Vip+ GABAergic Neuron 3
Oligodendrocyte
Non−neuronal Cell
LAMP5+ BMP2+ Layer 1 GABAergic Neuron
rawPCC
Sst+ GABAergic Neuron 5
VIP+ SCML4+ Layer 1−2 GABAergic Neuron
Vip+ GABAergic Neuron 2
Sncg+ GABAergic Neuron 4
Signaling Alveolar Epithelial Type 2
Schwann
OPALIN+ MAP6D1+ Layer 2−6 Oligodendrocyte
OPALIN+ LDLRAP1+ Layer 5−6 Oligodendrocyte
OPALIN+ FTH1P3+ Layer 2−6 Oligodendrocyte
OPALIN+ ENPP6+ Layer 3−6 Oligodendrocyte
Oligodendrocyte
Oligodendrocyte 1
Lamp5+ GABAergic Neuron 4
Lamp5+ GABAergic Neuron 2
Alpha Islet Cell
Azimuth Cell Types 2021
−log10(qvalue)
Overlap Percentage
1.00
0.75
0.50
0.25
0.00
B
Breast Cancer Bcell network top 100 central genes
scHumanNet
B
B Cell
Naive B Cell, Lambda Light Chain
Naive B Cell
Intermediate B Cell, Lambda Light Chain
Intermediate B Cell
Plasmacytoid Dendritic Cell
Naive B Cell, Kappa Light Chain
Myeloid Dendritic Type 2
Memory B Cell, Kappa Light Chain
Memory B Cell
Dendritic Cell
Conventional Dendritic Cell 2 2
CD8 T
Plasmacytoid Dendritic
SCINET
B
Naive B Cell, Lambda Light Chain
Naive B Cell
Plasma
Intermediate B Cell, Lambda Light Chain
Intermediate B Cell
B Cell
PVALB+ ISG20+ Layer 3−5 GABAergic Neuron
Naive B Cell, Kappa Light Chain
Memory B Cell, Lambda Light Chain
Memory B Cell, Kappa Light Chain
Memory B Cell
Classical Monocyte
CD4+ Effector Memory T 3
CD4+ Central Memory T 2
bigSCale2
Plasma
Plasma Cell
Plasmacytoid Dendritic
Oligodendrocyte
Neuroendocrine
Double−negative T
Double−negative T 2
Delta Islet Cell
CD8+ Naive T 2
AXL+ Myeloid Dendritic Cell
AXL+ Dendritic Cell
Conventional Dendritic Cell 1
Ciliated
Plasmablast
GRNboost2
CD16+ Monocyte
Plasma
Plasmacytoid Dendritic Cell
Proliferating Basal
Neuroendocrine
Layer 6 Car3+ Glutamatergic Neuron, Intratelencephalon−Projecting
FEZF2+ CFTR+ Layer 5−6 Glutamatergic Neuron
Double−negative T 2
Differentiating Basal
Dendritic Cell
Conventional Dendritic Cell 2 2
Classical Monocyte
CD8+ Effector Memory T 6
CD8+ Effector Memory T 5
B Cell
MetaCell
Plasma
Plasma Cell
Double−negative T
Double−negative T 2
Plasmacytoid Dendritic
Neuroendocrine
Gamma−Delta T 3
Double−negative T 1
Delta Islet Cell
CD8+ Naive T 2
Plasmacytoid Dendritic Cell
Ciliated
Capillary
Plasmablast
CD4+ Naive T
rawPCC
CD8 T
VIP+ SCML4+ Layer 1−2 GABAergic Neuron
SST+ PRRT4+ Layer 1−2 GABAergic Neuron
SST+ DNAJC14+ Layer 5−6 GABAergic Neuron
SST+ CLIC6+ Layer 1−2 GABAergic Neuron
SST+ C4orf26+ Layer 5−6 GABAergic Neuron
SST+ BEAN1+ Layer 5−6 GABAergic Neuron
Schwann
OPALIN+ ENPP6+ Layer 3−6 Oligodendrocyte
Oligodendrocyte 1
Lamp5+ GABAergic Neuron
LAMP5+ CRABP1+ Layer 5−6 GABAergic Neuron
LAMP5+ BMP2+ Layer 1 GABAergic Neuron
Delta Islet Cell
CD8+ Memory/Effector T
Azimuth Cell Types 2021
−log10(qvalue)
Overlap Percentage
1.00
0.75
0.50
0.25
0.00
C
Breast Cancer Myeloid network top 100 central genes
scHumanNet
Immune
TYROBP+ CD74+ Layer 1−6 Microglia
OLR1+ Classical Monocyte
Classical Monocyte
CD14+ Monocyte
Natural Killer Cell
Monocyte
Microglia / Perivascular Macrophage
Dendritic Cell
Conventional Dendritic Cell 2 1
CD56−dim Natural Killer
Plasmacytoid Dendritic
Macrophage
Proliferating Natural Killer
Innate Lymphoid Cell
SCINET
Monocyte
CD14+ Monocyte
Immune
OLR1+ Classical Monocyte
Dendritic Cell
Nonclassical Monocyte
Myeloid Dendritic Type 2
EREG+ Dendritic
Conventional Dendritic Cell 2 2
CD16+ Monocyte
Intermediate Monocyte
Conventional Dendritic Cell 2 1
Classical Monocyte
CD56−dim Natural Killer
Basophil/Mast 2
bigSCale2
CD4+ Proliferating T
Cycling
Proliferating NK/T
Proliferating Macrophage
Plasmablast
Proliferating Basal
CD8+ Proliferating T
Proliferating Natural Killer
GRNboost2
Proliferating Basal
Plasmacytoid Dendritic
Monocyte
Classical Monocyte
CD16+ Monocyte
Proliferating NK/T
Neuroendocrine
Layer 6 Glutamatergic Neuron, Intratelencephalon−Projecting 3
Layer 6 Car3+ Glutamatergic Neuron, Intratelencephalon−Projecting
FEZF2+ CFTR+ Layer 5−6 Glutamatergic Neuron
CD8+ Effector Memory T 5
CD4+ Central Memory T 2
CD4 T
B Cell
Alpha Islet Cell
MetaCell
TREM2+ Dendritic
Smooth Muscle
Proliferating Macrophage
Airway Smooth Muscle
Signaling Alveolar Epithelial Type 2
Serous
Intermediate Monocyte
Endothelial
Delta Islet Cell
Cycling
Classical Monocyte
CD8+ Memory/Effector T
Bronchial Vessel 2
Artery
Activated Stellate
rawPCC
Layer 6 Glutamatergic Neuron, Corticothalamic−Projecting
VIP+ SOX11+ Layer 2−5 GABAergic Neuron
VIP+ HTR3A+ Layer 1−2 GABAergic Neuron
VIP+ HS3ST3A1+ Layer 3−5 GABAergic Neuron
Vip+ GABAergic Neuron
THEMIS+ RGPD6+ Layer 5 Glutamatergic Neuron
THEMIS+ LINC01116+ Layer 5 Glutamatergic Neuron
Sncg+ GABAergic Neuron 2
Smooth Muscle
Lamp5+ GABAergic Neuron 1
LAMP5+ CARM1P1+ Layer 3 Glutamatergic Neuron
FEZF2+ FILIP1L+ Layer 5−6 Glutamatergic Neuron
AXL+ Myeloid Dendritic Cell
Artery
Airway Smooth Muscle
Azimuth Cell Types 2021
−log10(qvalue)
Overlap Percentage
1.00
0.75
0.50
0.25
0.00

Supplementary Figure 3. Identification of cell-type-specific genes by centrality in CGNs using scHumanNet and five other single-cell network inference methods. A–C. The top 100 hub genes in the networks specific for T cells (**A**), B cells (**B**), and myeloid cells (**C**) were tested for enrichment of cell-type-specific genes derived from the Azimuth celltype database. The results for networks obtained with SAVER imputation are not shown, as hub genes produced no cell-type-specific terms enriched for any cell type. The red vertical line corresponds to a *q*-value of 0.05 corrected with the Benjamini–Hochberg method.

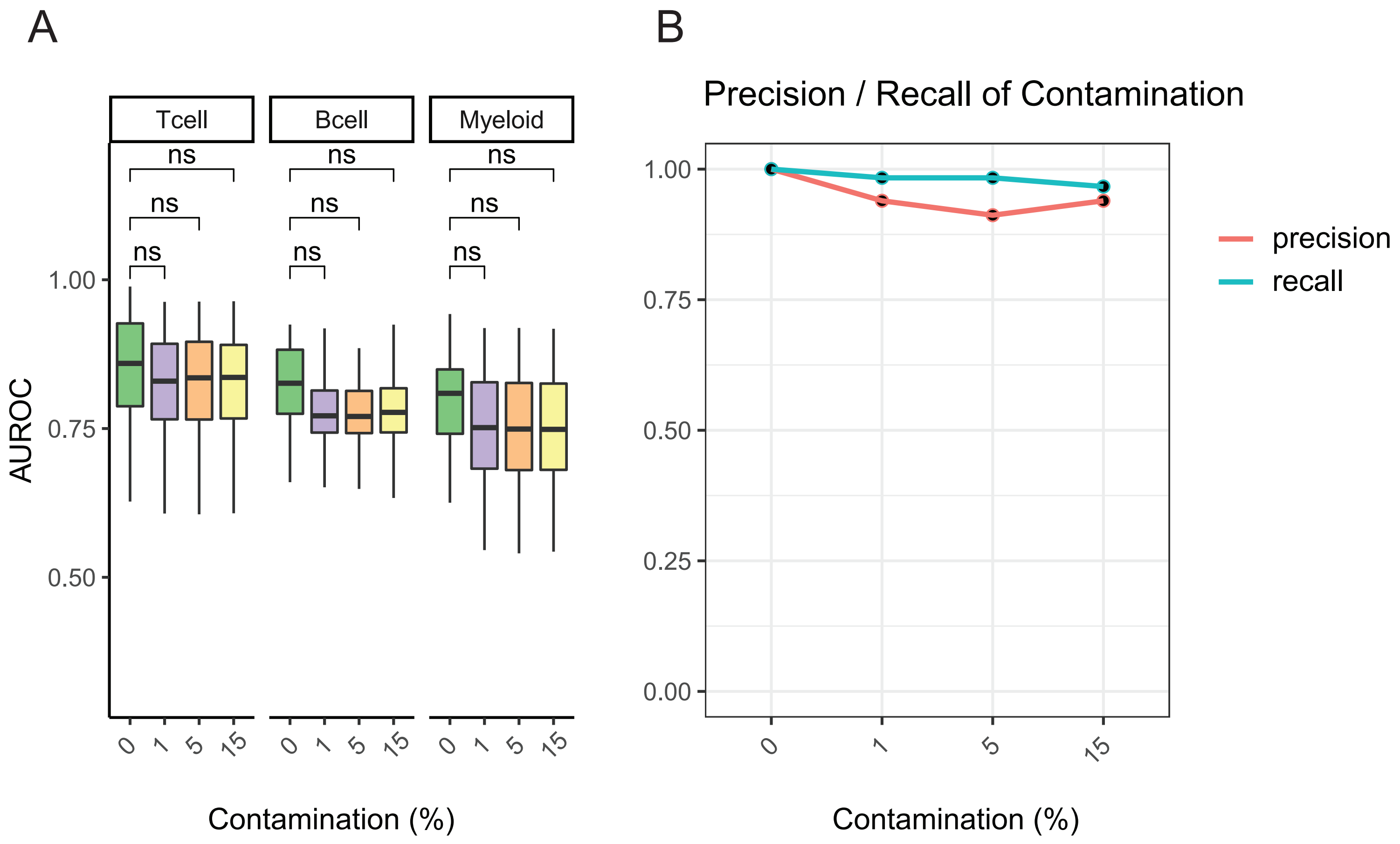
A
Tcell
Bcell
Myeloid
ns
AUROC
1.00
0.75
0.50
0
1
5
15
Contamination (%)
B
Precision / Recall of Contamination
1.00
0.75
0.50
0.25
0.00
precision
recall
0
1
5
15
Contamination (%)

Supplementary Figure 4. Robustness of cell-type-specificity of scHumanNet against contamination.

A. Contamination with 0 (zero) percent signifies the original network model constructed via scHumanNet pipeline. The given percentage of original cells for network construction was replaced with the same number of randomly selected cells from other immune cell subsets. Area under the receiver operating characteristic curve (AUROC) used to assess retrieval of cell-type-specific genes derived from the Azimuth cell type database by centrality in T, B, and myeloid cell-specific networks of breast cancer. AUROC scores for networks with contamination of other immune cell types are not significantly (ns) different from that of the original network that has no contamination (by two-tailed Mann Whitney U Test) **B.** Precision and recall calculation of the breast cancer T-cell scHumanNet with the same criteria for contamination. Precision and recall was calculated based on the T-cell specific genes collected from the Azimuth database.

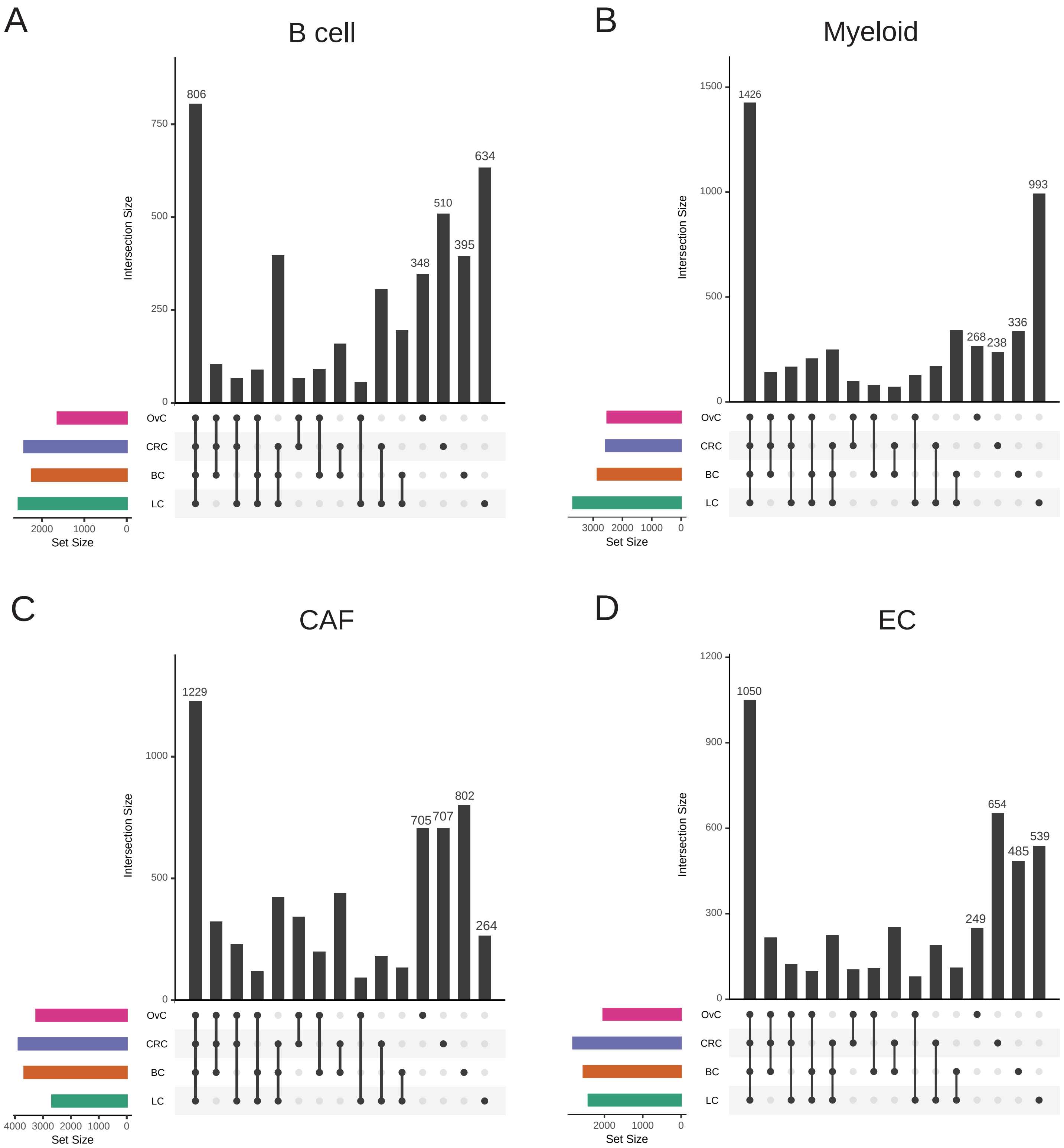
A
B cell
Intersection Size
806
634
510
395
348
OvC
CRC
BC
LC
Set Size
B
Myeloid
Intersection Size
1426
993
336
268
238
Set Size
C
CAF
Intersection Size
1229
802
705
707
264
Set Size
D
EC
Intersection Size
1050
654
539
485
249
Set Size

Supplementary Figure 5. Overlap of CGN nodes by scHumanNet among cancer types.

A–D. Upset plots for four cell types, including B cells (**A**), myeloid cells (**B**), CAFs (**C**), and ECs (**D**), showing overlap between CGN nodes among cancer types.

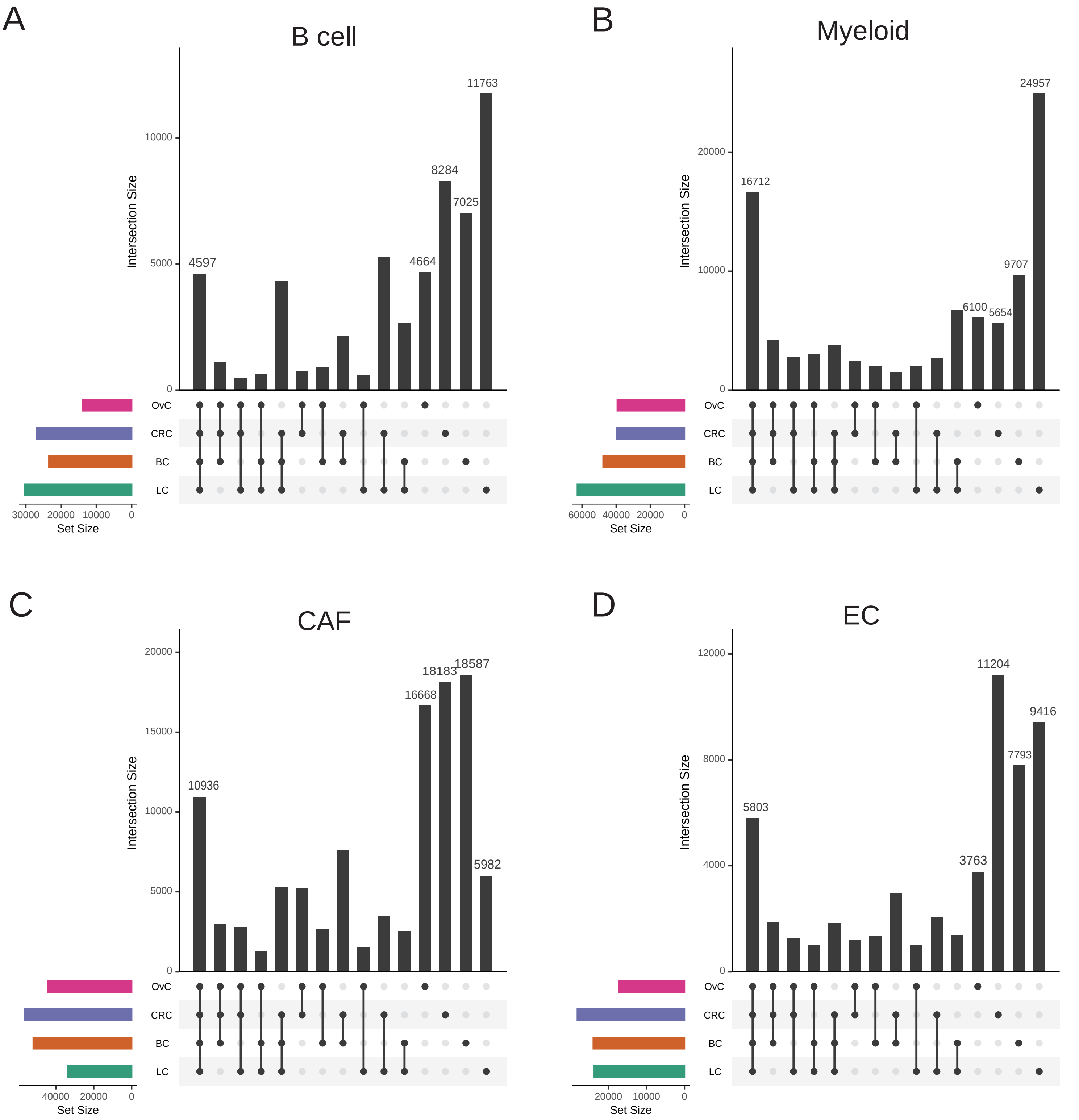
A
B cell
Intersection Size
4597
4664
8284
7025
11763
OvC
CRC
BC
LC
Set Size
B
Myeloid
16712
6100
5654
9707
24957
C
CAF
10936
16668
18183
18587
5982
D
EC
5803
3763
11204
7793
9416

Supplementary Figure 6. Overlap of CGN edges by scHumanNet among cancer types.

A–D. Upset plots for four cell types, including B cells (**A**), myeloid cells (**B**), CAFs (**C**), and ECs (**D**) showing overlap between CGN edges among cancer types.

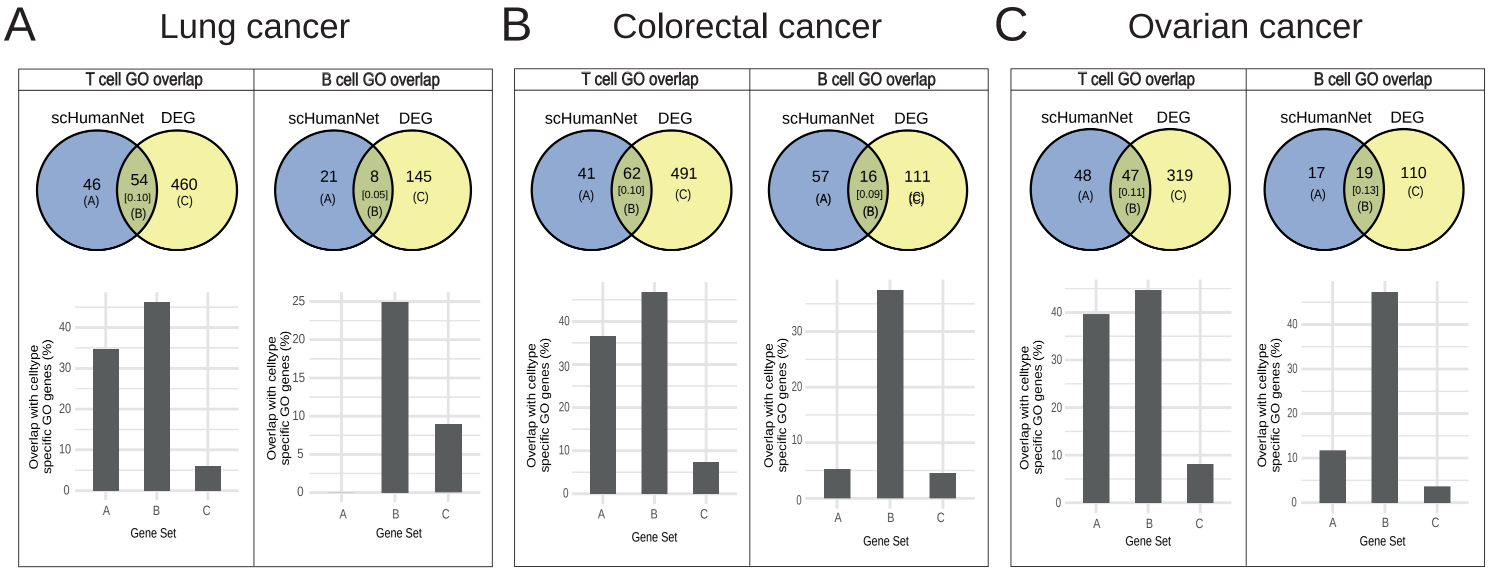
A
Lung cancer
B
Colorectal cancer
C
Ovarian cancer
T cell GO overlap
B cell GO overlap
scHumanNet
DEG
46 (A)
54 [0.10] (B)
460 (C)
21 (A)
8 [0.05] (B)
145 (C)
41 (A)
62 [0.10] (B)
491 (C)
57 (A)
16 [0.09] (B)
111 (C)
48 (A)
47 [0.11] (B)
319 (C)
17 (A)
19 [0.13] (B)
110 (C)
Overlap with celltype specific GO genes (%)
Gene Set
A
B
C

Supplementary Figure 7. Overlap of cell-type-specific genes predicted using gene expression and network centrality.

A–C. Venn diagram of T- or B-cell-specific genes predicted by significant DEGs and hubs in CGNs by scHumanNet for lung cancer (**A**), colorectal cancer (**B**), and ovarian cancer (**C**). The numbers in square brackets correspond to Jaccard indices. Overlap of genes specific for T- and B-cell functions was assessed for network and DEG-specific gene sets (set A and set C) and the intersection of both (set B).

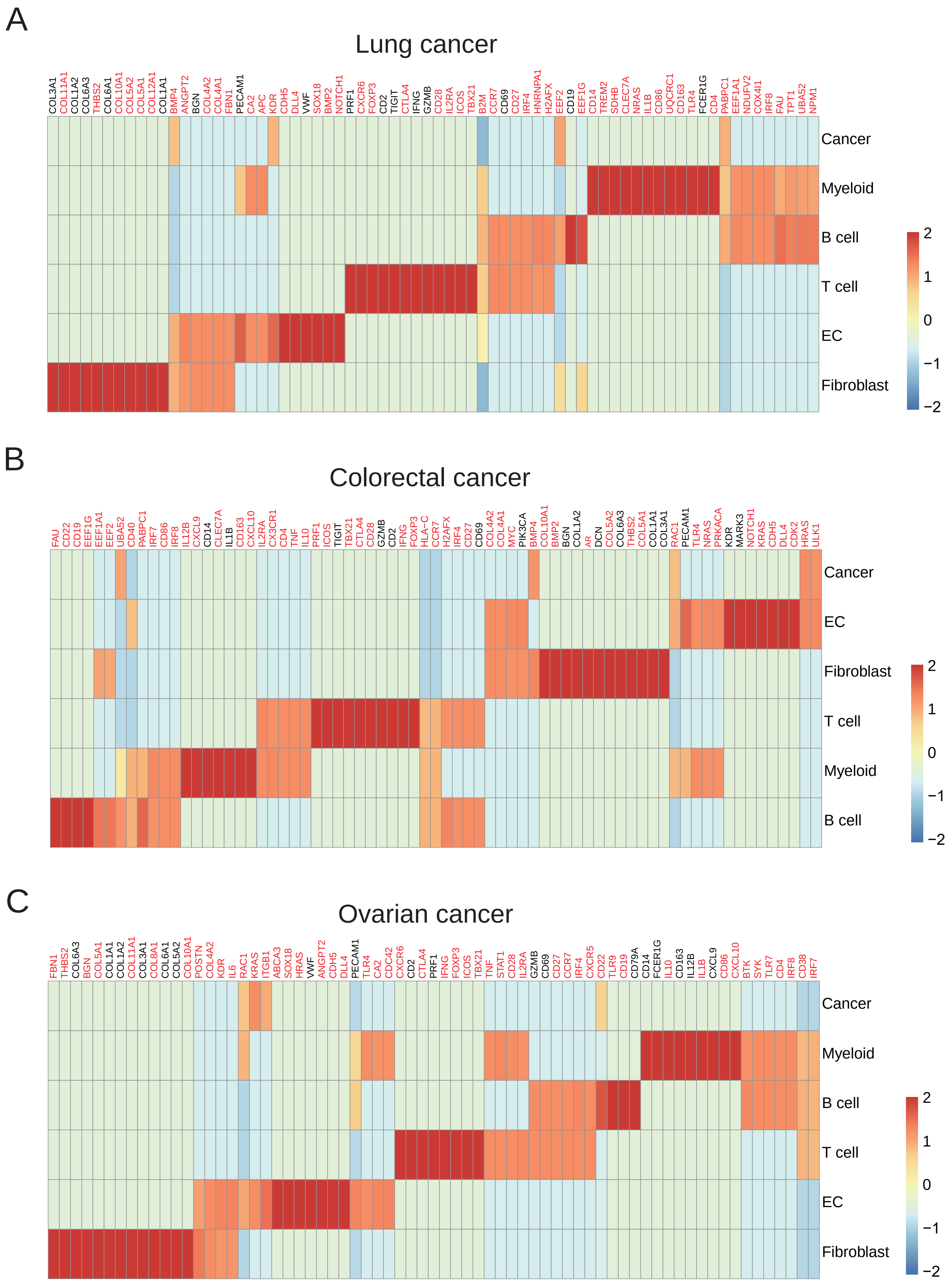
A
Lung cancer
COL3A1 COL11A1 COL1A2 COL6A3 THBS2 COL6A1 COL10A1 COL5A2 COL5A1 COL12A1 COL1A1 BMP4 ANGPT2 BGN COL4A2 COL4A1 FBN1 PECAM1 CA2 APC KDR CDH5 DLL4 VWF SOX18 BMP2 NOTCH1 PRF1 CXCR6 FOXP3 CD2 TIGIT CTLA4 IFNG GZMB CD28 IL2RA ICOS TBX21 B2M CCR7 CD69 CD27 IRF4 HNRNPA1 H2AFX EEF2 CD19 EEF1G CD14 TREM2 SDHB CLEC7A NRAS IL1B CD86 UQCRC1 CD163 TLR4 FCER1G CD4 PABPC1 EEF1A1 NDUFV2 COX4I1 IRF8 FAU TPT1 UBA52 NPM1
Cancer
Myeloid
B cell
T cell
EC
Fibroblast
2
1
0
−1
−2
B
Colorectal cancer
FAU CD22 CD19 EEF1G EEF1A1 EEF2 UBA52 CD40 PABPC1 IRF7 CD86 IRF8 IL12B CXCL9 CD14 CLEC7A IL1B CD163 CXCL10 IL2RA CX3CR1 CD4 TNF IL10 PRF1 ICOS TIGIT TBX21 CTLA4 CD28 GZMB CD2 IFNG FOXP3 HLA−C CCR7 H2AFX IRF4 CD27 CD69 COL4A2 COL4A1 MYC PIK3CA BMP4 COL10A1 BMP2 BGN COL1A2 DCN AR COL5A2 COL6A3 THBS2 COL5A1 COL1A1 COL3A1 RAC1 PECAM1 TLR4 NRAS PRKACA KDR MARK3 NOTCH1 KRAS CDH5 DLL4 CDK2 HRAS ULK1
Cancer
EC
Fibroblast
T cell
Myeloid
B cell
2
1
0
−1
−2
C
Ovarian cancer
FBN1 THBS2 COL6A3 BGN COL5A1 COL1A1 COL1A2 COL11A1 COL3A1 COL8A1 COL6A1 COL5A2 COL10A1 POSTN COL4A2 KDR IL6 RAC1 KRAS ITGB1 ABCA3 SOX18 HRAS VWF ANGPT2 CDH5 DLL4 PECAM1 TLR4 CA2 CDC42 CXCR6 CD2 CTLA4 PRF1 IFNG FOXP3 ICOS TBX21 TNF STAT1 CD28 IL2RA GZMB CD69 CD27 CCR7 IRF4 CXCR5 CD22 TLR9 CD19 CD79A CD14 FCER1G IL10 CD163 IL12B IL1B CXCL9 CD86 CXCL10 BTK SYK TLR7 CD4 IRF8 CD38 IRF7
Cancer
Myeloid
B cell
T cell
EC
Fibroblast
2
1
0
−1
−2

Supplementary Figure 8. Top 15 hub genes of the CGN generated by scHumanNet for cancers.

A–C. The top 15 hub genes were calculated as percentile ranks and scaled for lung cancer (**A**), colorectal cancer (**B**), and ovarian cancer (**C**). Genes highlighted in red were not included within the top 50 DEGs by Seurat's *FindMarkers()* function.

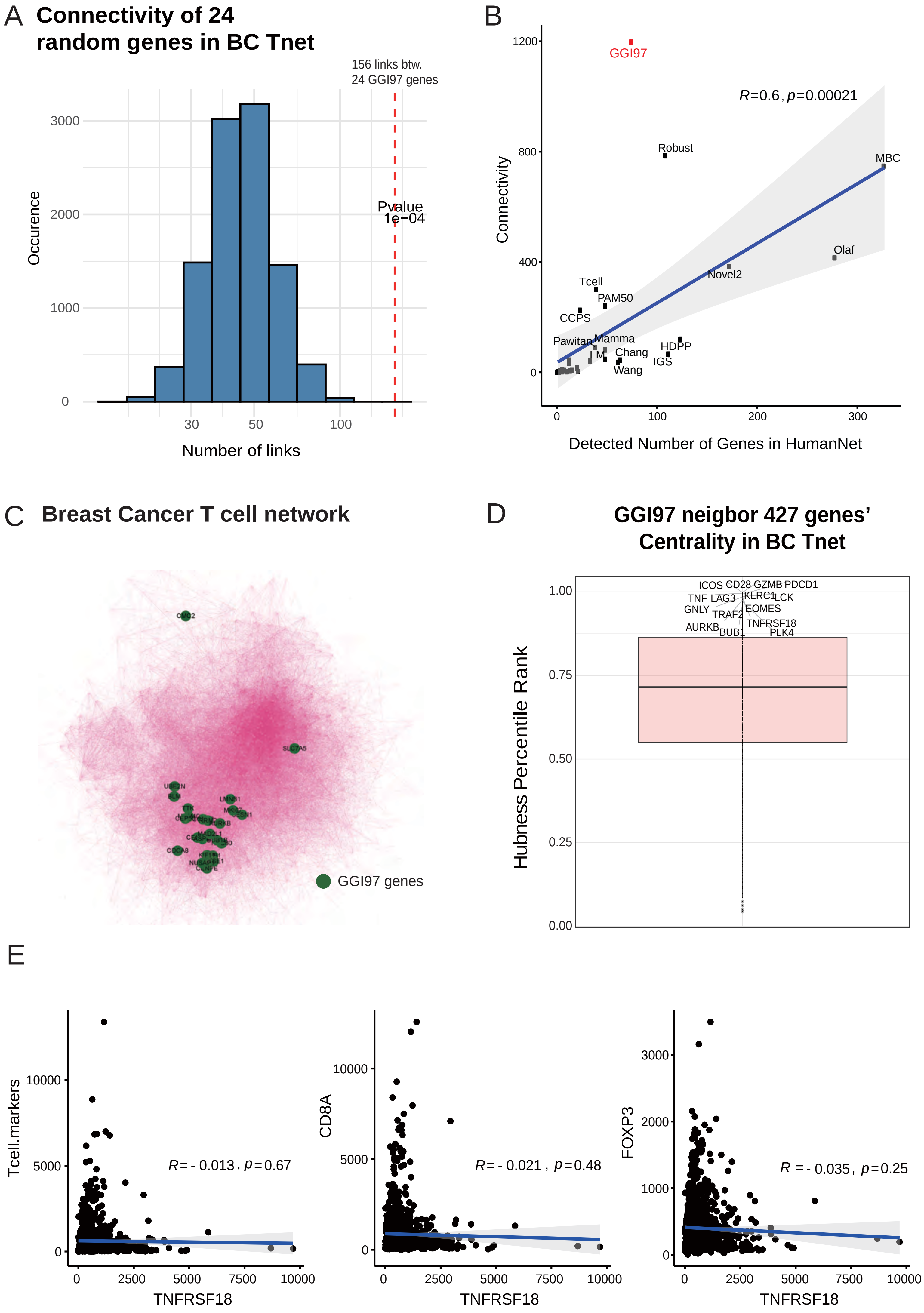

A
Connectivity of 24 random genes in BC Tnet
156 links btw. 24 GGI97 genes
Pvalue 1e−04
Occurence
3000
2000
1000
0
30
50
100
Number of links
B
GGI97
R=0.6, p=0.00021
Robust
MBC
Olaf
Novel2
Tcell
PAM50
CCPS
Pawitan
Mamma
LM
Chang
Wang
HDPP
IGS
Connectivity
1200
800
400
0
0
100
200
300
Detected Number of Genes in HumanNet
C
Breast Cancer T cell network
GGI97 genes
D
GGI97 neigbor 427 genes' Centrality in BC Tnet
ICOS CD28 GZMB PDCD1
TNF LAG3 KLRC1 LCK
GNLY TRAF2 EOMES
TNFRSF18
AURKB BUB1 PLK4
Hubness Percentile Rank
1.00
0.75
0.50
0.25
0.00
E
Tcell.markers
10000
5000
0
R= - 0.013, p=0.67
TNFRSF18
CD8A
10000
5000
0
R= - 0.021, p=0.48
TNFRSF18
FOXP3
3000
2000
1000
0
R = - 0.035, p=0.25
TNFRSF18
0
2500
5000
7500
10000

Supplementary Figure 9. Deconvolution of breast cancer signatures into cell types by scHumanNet.

A. Functional connectivity of 24 GGI genes detected in the T cell network for breast cancer by scHumanNet compared with the connectivity of 24 randomly selected genes. The *p*-value was calculated non-parametrically. **B.** Positive correlation between the number of signature genes detected in T-cell networks by scHumanNet and the number of connectivities. Within-group connectivity for each cell-type-specific network was normalized to network size (see Figure 4A). GGI97 showed high connectivity, despite only a moderate number of genes being detected (highlighted in red). **C.** Visualization of the entire breast cancer T-cell network with 2,611 nodes and 35,210 edges. GGI genes are highlighted in green. **D.** Percentile rank of first degree neighbors for all GGI97 genes. The top 15 genes were labeled. **E.** Correlation between TCGA-BRCA dataset with *TNFRSF18* (*GITR*) and T-cell-related signatures. TCGA-BRCA dataset was filtered for female samples and normalized using DESeq2. T-cell markers on the far right correspond to the mean expression of *CD3D, CD3E*, and *CD3G*. Pearson correlation coefficient (R) and Spearman correlation coefficient (ρ) were calculated.

Astrocyte

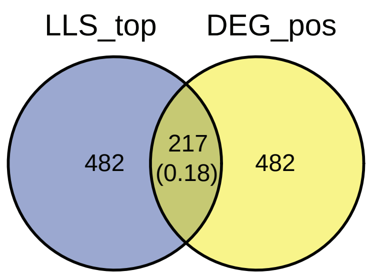

Oligodendrocytes

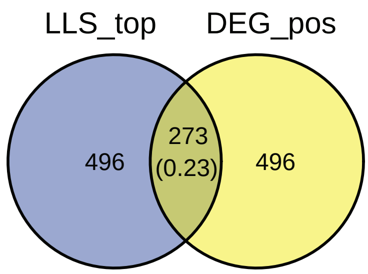

OPC

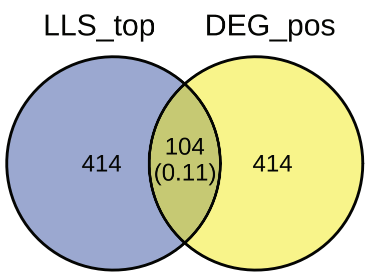

Endothelial

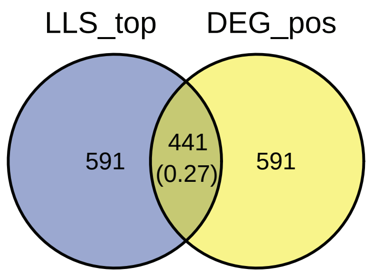

Microglia

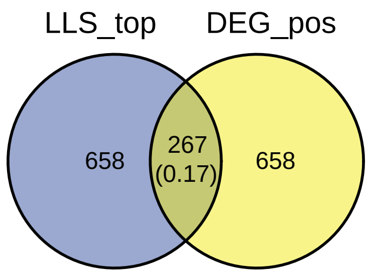

Excitatory

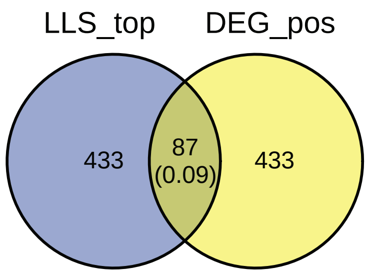

Inhibitory

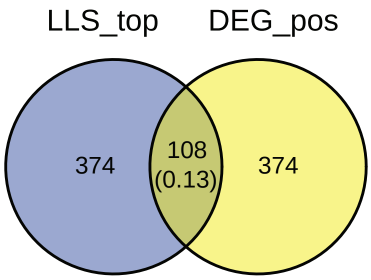

Supplementary Figure 10. Comparison of genes prioritized by DEGs and CGN hub genes in neuronal cell types.

For each cell type reported by Velmeshev *et al*. (2019), DEGs (Wilcoxon, FDR < 0.05, log fold change > 0.25) and hub genes in the CGN generated by scHumanNets were compared. The numbers in parenthesis correspond to Jaccard indices. OPC, oligodendrocyte progenitor cell.

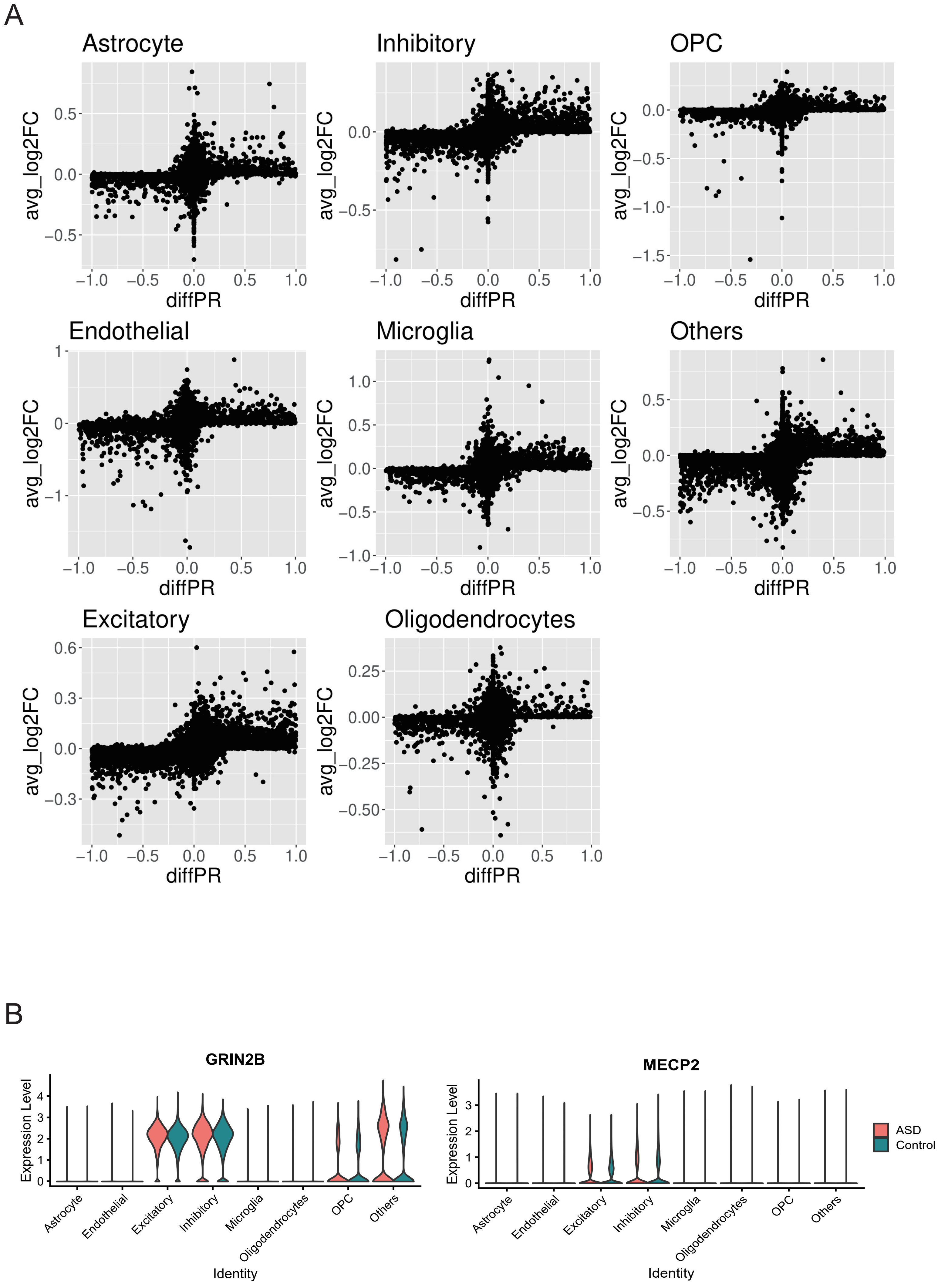
A
Astrocyte
Inhibitory
OPC
Endothelial
Microglia
Others
Excitatory
Oligodendrocytes
avg_log2FC
diffPR
B
GRIN2B
MECP2
Expression Level
Identity
Astrocyte
Endothelial
Excitatory
Inhibitory
Microglia
Oligodendrocytes
OPC
Others
ASD
Control

Supplementary Figure 11. Differential hubness analysis for CGNs between control and autism spectrum disorder (ASD) conditions using scHumanNet.

A. Evaluation of differential hubness for each gene derived from network analysis and log fold change derived from scRNA-seq expression data. **B.** Expression levels of *GRIN2B* and *MECP2* of ASD and healthy control samples are presented for each brain cell type.

A Distribution of genes > 0.7 PR in ASD scHumanNet

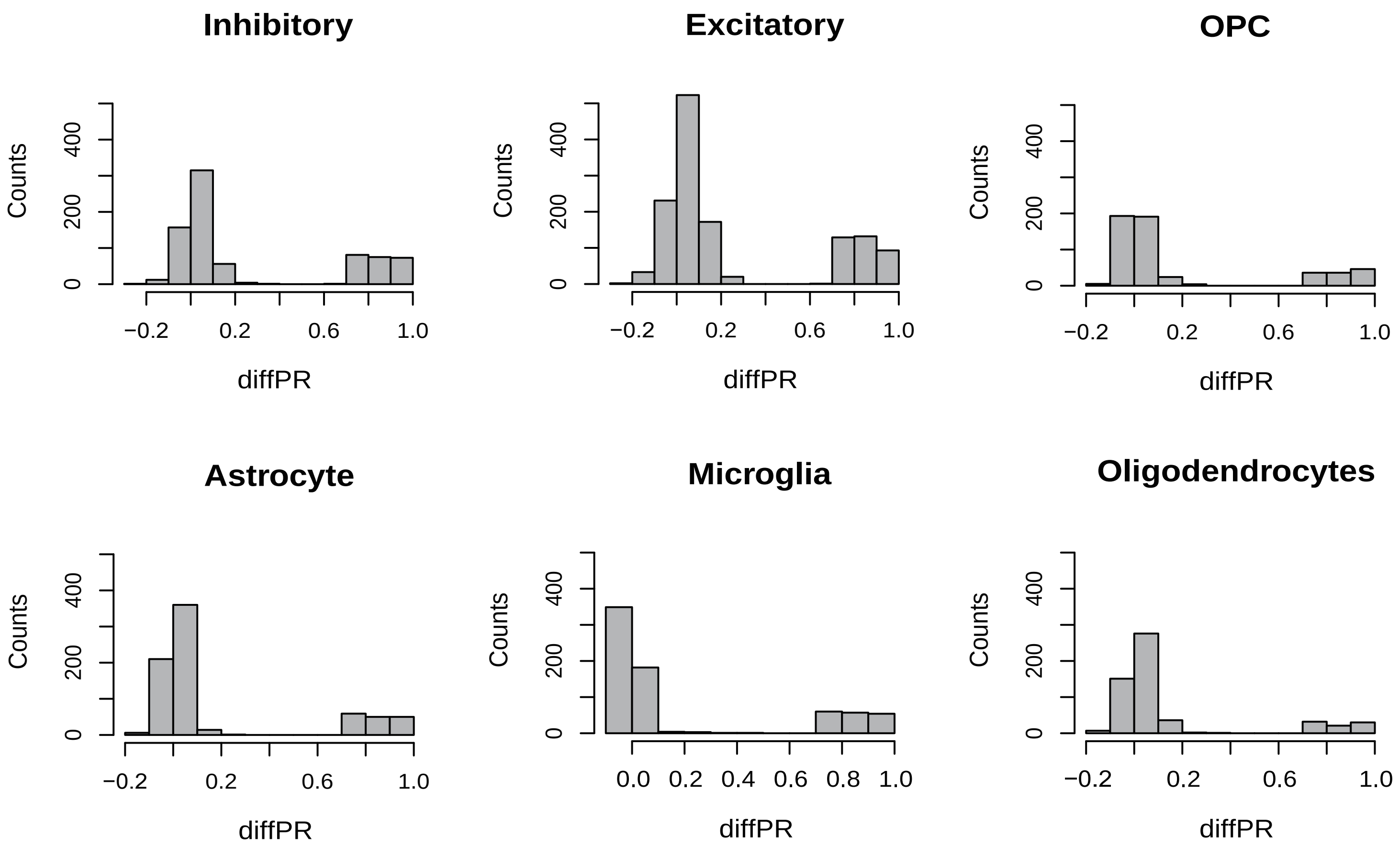

B Hallmark Pathways of genes > 0.7 PR in Control scHumanNet

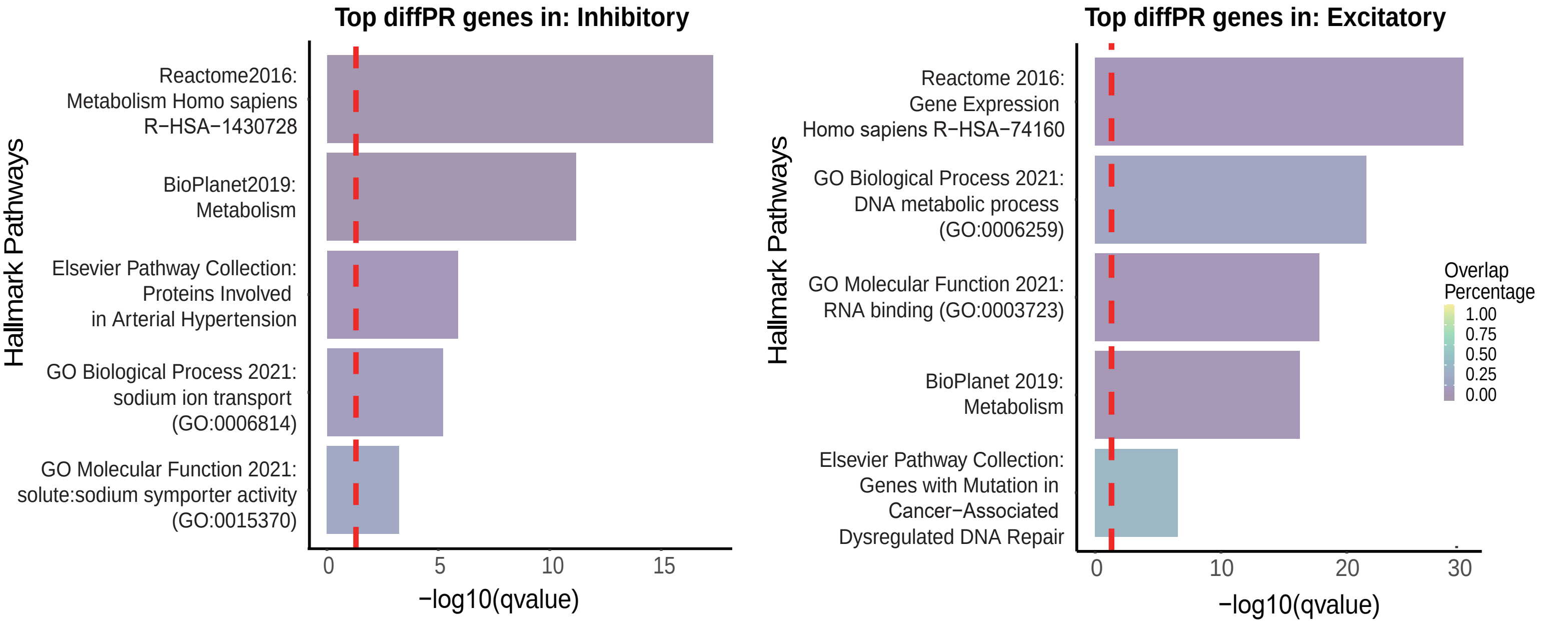

Supplementary Figure 12. Analysis of differential hubness genes between ASD and healthy controls.

A. Distribution of genes with > 0.7 centrality and > 0.7 differential hubness in ASD. **B.** Hallmark pathways associated with genes that have high centrality in ASD but low centrality in healthy controls based on five pathway databases (Reactome, BioPlanet, Elsevier Pathway Collection, GO Biological Process, GO Molecular Function). Pathways detected in inhibitory neurons (left) and excitatory neurons (right) are shown. The red vertical line corresponds to a q-value of 0.05 corrected with the Benjamini–Hochberg method.

Supplementary Table 1. List of genome grade index GGI97 signature genes

CMC2	TTK	NUSAP1	GTSE1
ASPM	MKI67	AURKB	RRM2
SLC7A5	KIF11	CENPE	MCM10
CEP55	CDCA8	NDC80	UBE2N
SESN1	CCNA2	BUB1B	MAD2L1
CENPA	BLM	LMNB1	BUB1
RACGAP1	BIRC5	CCNB1	CCNB2
CCNE2	CDC2	CDC20	CDC25A
KPNA2	MCM2	UBE2S	MYBL2
13CDNA73	BBS1	BM039	BRRN1
C20orf24	CCT5	CDCA3	CDK2
CDKN3	CENPF	CX3CR1	CYBRD1
DDX39	DKFZp762E1312	DLG7	DONSON
ESPL1	EXO1	FEN1	FLJ10156
FLJ20477	FLJ20641	FLJ21062	FLJ21827
FLJ23554	FOXM1	GMPS	H2AFZ
HMGB3	HMMR	HSMPP8	KIF20A
KIF2C	KIF4A	KNSL7	LAMB2
MARS	MELK	MLF1IP	NUDT1
OIP5	ORMDL2	PLK1	POLQ
PRC1	RNASEH2A	SHMT2	SIRT3
SPAG5	STARD13	STK6	TIMELESS
TPX2	TRIP13	TROAP	TTC10
ZWINT	MCM4		

Green: detected in Breast cancer T-cell CGN
Red: either deprecated, replaced or withdrawn from the NCBI database.

Supplementary Table 2. Number of nodes and edges in the CGNs used in network method comparison.

	# Nodes					# Edges				
	T cells	B cells	Myeloid cells	CAFs	ECs	T cells	B cells	Myeloid cells	CAFs	ECs
**scHumanNet**	2,611	2,242	2,855	3,665	2,550	35,120	23,430	47,678	51,851	23,994
**bigSCale2**	1,890	1,195	3,928	1,872	1,757	53,000	34,000	56,000	20,000	14,000
**SAVER**	1,115	797	2,172	1,009	1,283	32,000	16,000	35,000	22,000	26,000
**GRNboost2**	2,332	1,840	2,143	2,795	2,469	5,302	3,946	4,539	6,071	5,354
**MetaCell**	2,739	1,819	4,133	1,915	1,103	96,000	24,000	44,000	19,000	8,000
**rawPCC**	121	716	530	134	557	221	3,230	1366	167	1313

CAFs, cancer-associated fibroblasts; ECs, endothelial cells.

Supplementary Table 3. Number of genes and edges in CGNs inferred by scHumanNet for four cancer types.

Cancer type	Cell type	# Genes	# Edges
**Colorectal cancer**	T cell	2,521	31,768
	B cell	2,421	26,997
	Myeloid	2,568	39,794
	Endothelial	2,821	28,197
	Fibroblast	3,873	56,487
	Enteric glia	1,951	13,164
	Epithelial	3,204	61,057
	Mast	1,539	9,585
	Tumor	4,423	89,859
**Breast cancer**	T cell	2,611	35,210
	B cell	2,242	23,430
	Myeloid	2,855	47,678
	Endothelial	2,550	23,994
	Fibroblast	3,665	51,851
	Dendritic cell	1,996	24,236
	Mast	1,522	8,882
	Tumor	4,426	79,920
**Lung cancer**	T cell	2,726	35,933
	B cell	2,554	30,365
	Myeloid	3,687	62,810
	Endothelial	2,420	23,740
	Fibroblast	2,669	33,822
	Alveolar	2,839	31,910
	Epithelial	2,482	23,997
	Mast	1,862	13,053
	Tumor	5,075	111,043
**Ovarian cancer**	T cell	1,957	24,824
	B cell	1,633	13,806
	Myeloid	2,521	39,375
	Endothelial	2,033	17,204
	Fibroblast	3,238	44,083
	Tumor	3,977	72,159

Supplementary Table4. List of the 43 immune checkpoint molecule genes used in this study.

ADORA2A	BTLA	CD200	CD200R1
CD244	CD27	CD274	CD276
CD28	CD40	CD40LG	CD80
CD86	CEACAM1	CTLA4	HAVCR1
HAVCR2	ICOS	ICOSLG	IDO1
IL2RB	KIR3DL1	LAG3	LAIR1
LGALS3	NECTIN2	PDCD1	PDCD1LG2
PVR	SLAMF1	TIGIT	TNFRSF12A
TNFRSF14	TNFRSF18	TNFRSF25	TNFRSF4
TNFRSF9	TNFSF14	TNFSF18	TNFSF4
TNFSF9	VSIR	VTCN1	

Supplementary Table 5. Number of genes and links in CGNs inferred by scHumanNet for autism spectrum disorder and healthy controls.

Condition	Cell type	# Genes	# Links
**Autism spectrum disorder**	Astrocyte	2,505	26,610
	Excitatory	4,488	68,448
	Inhibitory	2,597	23,992
	Microglia	2,387	32,270
	Endothelial	2,394	35,789
	OPC	1,784	14,017
	Oligodendrocyte	1,860	11,629
	Others	2,532	27,807
**Control**	Astrocyte	2,549	27,810
	Excitatory	5,731	117,415
	Inhibitory	3,164	36,348
	Microglia	2,375	31,190
	Endothelial	2,626	40,769
	OPC	2,154	18,613
	Oligodendrocyte	2,203	16,660
	Others	3,234	50,535

OPC, oligodendrocyte progenitor cell.
